# Impact of Hydrophobic, Hydrophilic, and Mucus-Binding Motifs on the Therapeutic Potential of Ceftazidime Analogs for Pulmonary Administration

**DOI:** 10.3390/antibiotics14020177

**Published:** 2025-02-11

**Authors:** Kyle D. Apley, Stephanie N. Johnson, Jian Qian, Indeewara Munasinghe, Jennifer R. Klaus, Srilaxmi M. Patel, Kathryn E. Woods, Samalee Banerjee, Josephine R. Chandler, Chamani Perera, Nathalie Baumlin, Matthias Salathe, Cory J. Berkland

**Affiliations:** 1 Department of Pharmaceutical Chemistry, University of Kansas, Lawrence, KS 66045, USA; 2 Synthetic Chemical Biology Core Laboratory, University of Kansas, Lawrence, KS 66045, USA; 3 Department of Molecular Biosciences, University of Kansas, Lawrence, KS 66045, USA; 4 Department of Internal Medicine, University of Kansas Medical Center, Kansas City, KS 66160, USA; 5 Department of Chemical and Petroleum Engineering, University of Kansas, Lawrence, KS 66045, USA; 6 Bioengineering Graduate Program, University of Kansas, Lawrence, KS 66045, USA

**Keywords:** antibacterial(s), drug design, drug transport, epithelial permeability, pulmonary drug delivery

## Abstract

**Background/Objectives**: The pulmonary administration of antibiotics can be advantageous in treating pulmonary infections by promoting high intrapulmonary drug concentrations with reduced systemic exposure. However, limited benefits have been observed for pulmonary administration versus other administration routes due to its rapid clearance from the lung. Here, the effects of structural modifications on the epithelial permeability and antibacterial potency of a third-generation cephalosporin were investigated to improve the understanding of drug properties that promote intrapulmonary retention and how they may impact efficacy. **Methods**: Ceftazidime was modified by attaching 18 hydrophobic, hydrophilic, and mucus-binding motifs to the carboxylic acid distant from the beta-lactam by amidation. Epithelial permeability was investigated by drug transport assays using human bronchial epithelial air–liquid interface cultures. Antibacterial potency was determined by microtiter MIC assays with *B. pseudomallei*, *P. aeruginosa*, *E. coli*, and *S. aureus*. **Results**: A 40–50% reduction in the transepithelial transport rate was exhibited by two PEGylated ceftazidime analogs (mPEG8- and PEG5-pyrimidin-2-amine-ceftazidime) and n-butyl-ceftazidime. An increase in the transport rate was exhibited by four analogs bearing small and hydrophobic or negatively charged motifs (n-heptane-, phenyl ethyl-, glutamic acid-, and 4-propylthiophenyl boronic acid-ceftazidime). The antibacterial potency was reduced by ≥10-fold for most ceftazidime analogs against *B. pseudomallei*, *P. aeruginosa*, and *E. coli* but was retained by seven ceftazidime analogs primarily bearing hydrophobic motifs against *S. aureus*. **Conclusions**: The covalent conjugation of PEGs with MW > 300 Da reduced the epithelial permeability of ceftazidime, but these modifications severely reduced antibacterial activity. To improve the pulmonary retention of antibiotics with low membrane permeability, this work suggests future molecular engineering studies to explore high-molecular-weight prodrug strategies.

## 1. Introduction

The lungs are particularly susceptible to infection due to the constant exposure to pathogens from the environment [1,2,3]. Lung infections treated via oral or injected antibiotics may exhibit poor response rates due to their limited penetration into infected lung tissue, rapid elimination of the therapeutic, and dose-limiting side effects [4,5]. The accessibility and large surface area of the lungs that leaves them liable to infections also provides an opportunity for inhaled antibiotics which have been used since the 1940s to treat chronic airway infections [2,5,6,7]. The administration of antibiotics directly to the lungs is advantageous because it allows for high pulmonary epithelial lining fluid (PELF) concentrations with lower systemic exposure which limits the potential for adverse side effects [1,2,5,6,7,8,9,10]. However, drugs can be rapidly eliminated from the lungs via mucociliary clearance, enzymatic degradation, macrophage uptake, or absorption [11,12]. In fact, many small molecule drugs delivered directly to the lungs exhibit relatively rapid absorption and near complete systemic bioavailability [12,13,14,15,16,17]. This phenomenon hinders the time or concentration-based killing of lung pathogens by antimicrobials. Thus, physiochemical factors that avoid these clearance mechanisms may enhance the safety and efficacy of inhaled antibiotics [1].

To promote retention in the lungs, some have suggested that drugs need to have a high molecular mass (>600 Da) [9,10]. Additionally, hydrophilic drug properties may promote retention in PELF or mucus and prevent transcellular absorption pathways, but hydrophobic drug properties may slow dissolution and facilitate drug retention in lipid membranes [18,19,20]. Antibiotics are typically designed for absorption after oral administration or for solubility in injectable formulations, which may select for drug properties that are not ideal for lung retention after inhalation.

**Scheme 1:**
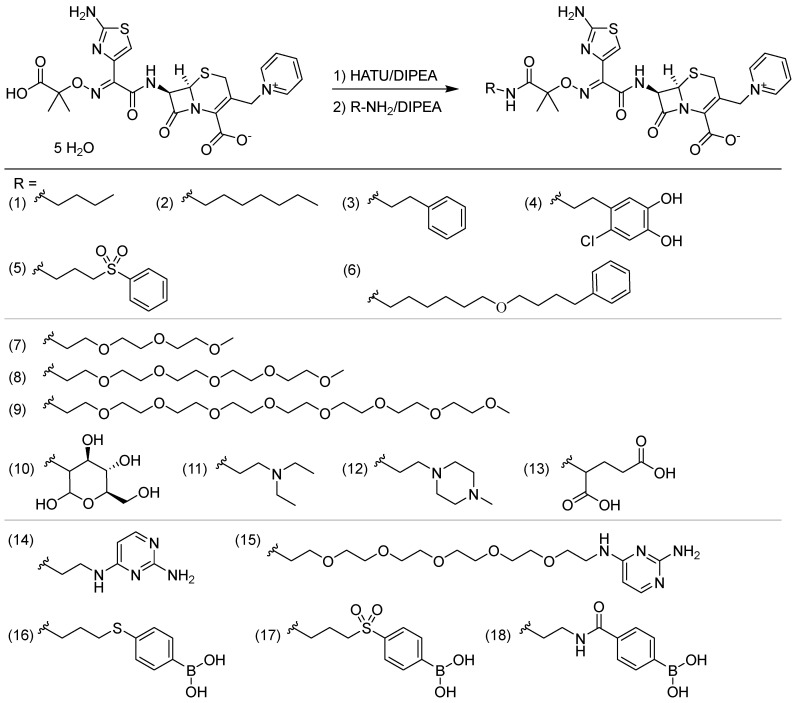
Synthesis of ceftazidime analogs. R groups are categorized as hydrophobic (**top**), hydrophilic (**middle**), and mucus-binding (**bottom**).

It may be useful to purposefully design antibiotics that exhibit properties to persist in the lungs. To explore this concept, ceftazidime was selected as the core antibiotic pharmacophore for chemical modifications (Scheme 1). Ceftazidime exhibits a wide spectrum of activities against Gram-negative aerobic bacteria with the coverage of some Gram-positive bacteria, and it possesses a carboxylic acid handle distant from the warhead β-lactam for modification [21,22]. In addition, ceftazidime has been investigated for pulmonary administration in both animal models and humans but is rapidly absorbed from the lung, requiring intensive dosing schedules to maintain the desirable trough concentrations [23,24,25,26]. Therefore, we synthesized multiple analogs of ceftazidime with appended structural motifs to alter the transport characteristics. The potential for increased persistence in the lung was assessed using an in vitro transport assay, and antibacterial activity was determined by a minimum inhibitory concentration (MIC) assay.

## 2. Results

### 2.1. Ceftazidime Analog Synthesis

Ceftazidime analogs were synthesized as shown in Scheme 1. The syntheses were completed in two groups designated as set 1 (**1, 8, 9, 11, 15**) and set 2 (**2–7, 10, 12–14, 16–18**). The ceftazidime analogs were designed to modulate transport by increasing hydrophobicity (**1–6**), hydrophilicity (**7–13**), or mucus-binding (**14–18**). Conjugated motifs to increase hydrophobicity included linear alkyl chains with and without terminal phenyl groups with the aim of promoting non-specific binding or evoking partial insertion into lipid membranes to limit transport across the epithelium [18,19]. Conjugated motifs to increase hydrophilicity included PEG of varying lengths, a monosaccharide, and several charged moieties with the aim of increasing size and reducing membrane permeability [20]. The mucus-binding 2,4-diaminopyrimidine group in (**14**) and (**15**) was previously identified by Witten and colleagues [27] whereas the arylboronic acid groups in (**16**), (**17**), and (**18**) were selected to bind mucus glycoproteins through cis-diol exchange at the physiological pH [28,29]. The diversity of the conjugated motifs was selected in order to determine the effects of size and hydrophobicity/hydrophilicity on epithelial transport and antibacterial activity.

### 2.2. Lung Tissue Culture Transport Assays

The transport of ceftazidime analogs was investigated in a model of the lung epithelium consisting of a human bronchial epithelial cell air–liquid interface culture on Transwells. The compound flux across the epithelial cell layer was measured by quantifying the compound in the media on the basal side of the membrane over 24 h (Figures S1 and S2) then converting into to apparent permeability (P_app_) [30]. Transport was investigated in this manner with two sets of ceftazidime analogs that utilized human bronchial epithelial cells (HBECs) from different donors, and transepithelial electrical resistance (TEER) was used to assess the integrity of the cell monolayers. Different donors for the primary HBECs used in the cultures were necessary because of the time between the completion of the synthesis of the first and second set of compounds. In set 1, the TEER values exceeded 500 Ωcm^2^ for all three donors supporting the creation of complete cell layers with tight junctions in each Transwell (Table S1) [31]. In set 2, the TEER values varied amongst the four donors with cultures from one donor (L8) exhibiting TEER values < 500 Ωcm^2^ for all Transwells (Table S2). All data points from this donor were excluded to improve the accuracy as TEER values less than 500 Ωcm^2^ exhibit the increased paracellular transport of hydrophilic molecules inversely correlated to the TEER value [32].

Between the two sets, the P_app_ of ceftazidime was not statistically different with *p* = 0.38 (Table 1 and Table S3). In set 1, the P_app_ was significantly (*p* < 0.05) lower for compounds (**1**), (**9**), and (**15**) compared to ceftazidime (Table 1 and Table S4). None of the compounds tested in set 2 had significantly lower P_app_s than ceftazidime although compounds (**14**) and (**18**) trended lower (Table 1 and Table S4). In contrast, the P_app_s for compounds (**2**), (**3**), (**13**), and (**16**) in set 2 were significantly greater than ceftazidime (Table 1 and Table S5).

**Figure 1:**
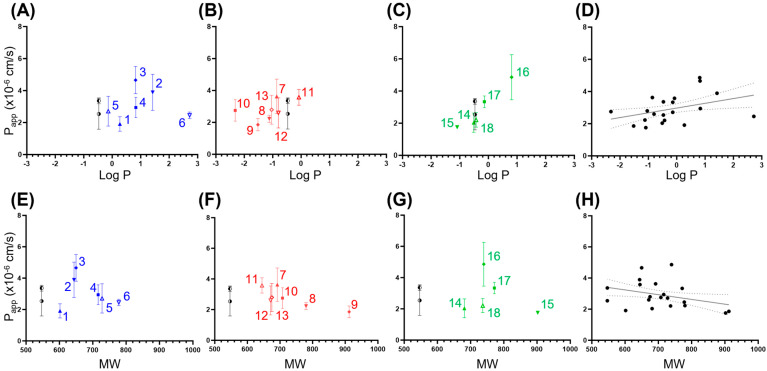
Plots of ceftazidime analog P_app_ as a function of the predicted LogP (**A**–**D**) and MW (**E**–**H**). The error bars represent the standard deviation. The black data points are ceftazidime. (**A**,**E**) depict the analogs designed to have increased hydrophobicity in blue. (**B**,**F**) depict the analogs designed to have increased hydrophilicity in red. (**C**,**G**) depict the compounds designed to bind mucus in green. (**D**,**H**) depict the correlation between the respective factor and P_app_ with the dotted line as bounds of the 95% CI for the correlation.

The correlation between lipophilicity and molecular weight on HBEC transport was examined to assess if either property may drive the increased apical retention of ceftazidime (Figure 1). Lipophilicity was estimated by averaging the predicted LogP values from atom-based, fragment-contribution, and property-dependent computational methods to minimize respective biases. Specifically, the computational methods MLogP, ALogP, ALogPs, and XLogP2 were used and accessed from the Virtual Computational Chemistry Laboratory [33]. A positive correlation between P_app_ and the predicted LogP approached statistical significance (*p* = 0.055, r = 0.436) (Figure 1D). A negative correlation between P_app_ and MW was not statistically significant (*p* = 0.112, r = −0.366), although two of the three ceftazidime analogs with significantly reduced P_app_ values were the highest MW compounds evaluated (Figure 1H). The correlation between P_app_ and eight other molecular descriptors was evaluated but none were statistically significant (Figure S3) [34].

### 2.3. MIC Determination of Ceftazidime Analogs

Each analog had reduced antibacterial activity against three or all four of the pathogens compared to ceftazidime (Table 1). The reduction in antibacterial activity against the Gram-negative *B*. *pseudomallei* and *P*. *aeruginosa* was comparable for each ceftazidime analog. Only the analogs bearing the polar, cyclic, and uncharged (**4**, **10**, **18**) motifs had MIC values <10× (60 µg/mL) that of ceftazidime for these two pathogens. For Gram-negative *E. coli*, only the analog bearing the polar, cyclic, and uncharged (**10**) had MIC values <10× (4 µg/mL) that of ceftazidime, but the analogs bearing the small, polar and neutral or anionic (**4**, **5**, **13**, **18**) had MIC values <15 µg/mL. Antibacterial activity was best retained against the Gram-positive *S*. *aureus* by the ceftazidime analogs. The analogs bearing hydrophobic (**1**–**6**), mono-cationic (**11**), and phenylboronic acid (**16**–**18**) motifs had MIC values <3× (40 µg/mL) that of ceftazidime. Ceftazidime analogs bearing the larger PEG (**8**, **9**) and doubly cationic (**12**, **14**) motifs exhibited the poorest antimicrobial activity with MICs ≥100 µg/mL for each pathogen tested.

## 3. Discussion

Ceftazidime will reach concentrations in the PELF above the MIC for relevant respiratory pathogens when delivered via injection, but has a low penetration ratio (PELF-to-total plasma concentration) in the range of 20–30% [4,35]. The low penetration ratio is compounded by the short circulating half-life of ceftazidime in humans of 1.9 h, requiring frequent IV boluses or continuous infusion to retain effective concentrations in the PELF [36]. Ceftazidime is highly water-soluble with a LogP = −1.60, suggesting that it may be retained in the hydrophilic mucus of the respiratory tract following pulmonary administration [24,37]. However, its low molecular weight likely results in its rapid absorption into systemic circulation by paracellular diffusion through the lung epithelium [24,26,35].

Despite the reduced antibacterial potency, ceftazidime analogs bearing butyl, mPEG_8_, or PEG_5_-pyrimidin-2-amine groups had significantly slower transport across a model lung epithelial cell layer compared to ceftazidime. Ceftazidime analogs bearing mPEG_5_, ethyl pyrimidine, and boronic amide groups trended towards slower transport. The mechanism driving the reduced P_app_ of (**1**) bearing an N-butyl group is not entirely clear as the N-heptane bearing (**2**) trended towards having a higher permeability than ceftazidime, and the retention on the apical side of the epithelium by partial insertion into the plasma membrane would be expected to be greater for ceftazidime analogs with larger hydrophobic groups [38]. The reduced permeability of the mPEG_8_ bearing (**9**) and the PEG_5_-pyrimidin-2-amine bearing (**15**) is likely driven by a reduced diffusion rate due to the increase in hydrodynamic radii. Based on MW, the theoretical diffusion rates for (**9**) and (**15**) are estimated to be 20% less than that of ceftazidime using the Stokes–Einstein equation for a spherical particle [39]. The actual decrease in diffusion rate is likely greater than approximated here as the long-chain mPEG_8_ and PEG_5_-pyrimidin-2-amine groups are expected to form an ellipsoid particle which has a slower diffusion rate for the same molecular volume [40]. This reduction in diffusion rate accounts for a majority of the 40–50% reduction in P_app_ observed for these compounds.

As (**9**) and (**15**) both have similar MWs and P_app_s, the mucus-binding effects of the 2,4-diaminopyrimidine group on (**15**) did not appear to contribute significantly to this model system. The 2,4-diaminopyrimidine bearing (**14**) and the phenylboronic acid bearing (**16**–**18**) did not exhibit reduced P_app_s compared to ceftazidime either. This was unexpected as HBEC ALI cultures are capable of mucus secretion and the 2,4-diaminopyrimidine group is known to bind mucus [27].

The relationship between the predicted LogP and apparent permeability for the ceftazidime analogs was not as strongly correlated as in prior transepithelial transport studies using HBEC ALI cultures [41,42,43]. This may be due to the ceftazidime analogs with hydrophobic modifications being amphipathic and not as membrane permeable as the predicted LogP values suggest. As ceftazidime is polar and has limited membrane permeability, transcellular transport is likely a minor contributor to the transepithelial flux for all ceftazidime analogs.

Molecular mechanisms to increase ceftazidime persistence in the lung after inhalation are primarily limited to reducing the diffusion rate by increasing MW, specific binding events (ex. mucus-binding), or partial insertion into a plasma membrane. Substantially increasing persistence in the lung by increasing the MW of antimicrobials through irreversible conjugation to hydrophilic polymers is not likely to be a suitable strategy as the antimicrobial potency was greatly diminished. Increasing lung persistence by the latter two approaches will reduce the percentage of free drug in solution, requiring larger doses, but could increase the dosing interval. Alternatively, prodrug and slow-release particle approaches may be a faster path towards antibiotics with improved lung retention as the antibacterial potency would not be a barrier [44,45,46]. However, both prodrug and slow-release particle formulation strategies would primarily increase the dosing interval and are less likely to reduce the total dose as the half-life of the released drug in the lung would be the same. These limitations could hinder the benefit of pulmonary administration for antimicrobials with dose-dependent or exposure-associated toxicities [47].

Amongst the four pathogens tested, the activity against the Gram-positive *S. aureus* was affected the least. Whereas the reduced binding affinity for the targeted penicillin-binding proteins can hamper antibacterial activity in both Gram-positive and Gram-negative bacteria, the severe loss of antimicrobial activity in the Gram-negative bacteria suggests that the conjugated motifs result in the reduced accumulation in the periplasmic space due to the hindered outer membrane permeability or increased efflux [48,49]. From these results, it may be more achievable to investigate the development of covalently modified antimicrobial analogs for the treatment of Gram-positive bacterial infections if motifs similar in size and physicochemical properties to (**1**–**3**, **5**, **17**) are identified that strongly promote pulmonary retention.

## 4. Materials and Methods

### 4.1. Ceftazidime Analog Synthesis General Method

A solution of ceftazidime pentahydrate (200 mg, 0.314 mmol, 1 eq.) was prepared in dry dimethylformamide (16 mL) containing dry N,N-diisopropylethylamine (DIPEA) (190 µL, 1.10 mmol, 3.5 eq.) in a 50 mL round bottomed flask. Solid hexafluorophosphate azabenzotriazole tetramethyl uronium (HATU) (142.4 mg, 0.471 mmol, 1.2 eq.) was slowly added to the above solution and the container was then sealed and purged with Ar. The solution was stirred for 1 h under an Ar atmosphere. The color of the solution changed from light yellow to dark red with no precipitation. The corresponding amine (0.408 mmol, 1.3 eq.) was then added, followed by the injection of dry DIPEA (190 µL, 1.10 mmol, 3.5 eq.). The reaction solution was stirred for 15 h at room temperature under Ar. The purification was carried out by reverse phase HPLC using a prep Waters XBridge C18 5 µm column (Column ID: 19 mm × 150 mm, Waters Corporation, Milford, MA, USA) or prep HILIC 5 µm column (Column ID: 19 mm × 250 mm). Fractions containing the desired compound were combined and lyophilized to give a white to yellow solid. Its identity was confirmed by ^1^H NMR, ^13^C NMR, and HRMS (ESI^+^). The regioselectivity of the amidation was confirmed by 2D NMR.

### 4.2. Cell Culture and Transport Assays

The cell culture was performed as previously described [50,51,52]. Briefly, lung tissue was obtained from organ donors whose lungs were rejected for transplant and recovered for research by the Life Alliance Organ Recovery Agency at the University of Miami, FL, the LifeCenter Northwest in Seattle, WA, and the Midwest Transplant Network in Kansas City, KS. Human bronchial epithelial cells (HBECs) were expanded, then re-differentiated on Transwells (Corning, Corning, NY, USA) at the air–liquid interface (ALI) for a minimum of 28 days, after which the experiments were performed. A total of 50 μL of dissolved compound at approximately 5 mg/mL were added apically and 500 μL of basolateral media (ALI media) was collected at 1 h, 2 h, 4 h, 8 h, and 24 h and replaced with 500 μL of fresh media. At 24 h, 500 μL of Dulbecco’s phosphate-buffered saline was added apically to record the transepithelial electrical resistance (TEER) using the EVOM system (World Precision Instruments Inc., World Precision Instruments, Sarasota, FL, USA) at room temperature and collected for analysis. All collections were frozen at −80 °C upon analysis. Transwells with HBECs were frozen at −80 °C after the 24 h collection. The quantification of the ceftazidime analogs in the stock solution and media was performed by RP-HPLC-UV.

### 4.3. Data Analysis and Statistics

The rate of drug transport from the apical side to the basal media was compared by calculating the apparent permeability coefficient (P_app_, cm/s) [30]:

P_app_ = (dQ/dt) × (1/(A × C_0_)) (1)

where dQ/dt is the solute flux, determined by the slope at t = 0 of a non-linear regression of the form y = a(1 − e^−kt^) fit to the cumulative transport data with mg of compound on the y-axis and time (t) on the x-axis. A is the surface area (1.12 cm^2^), and C_0_ is the concentration of the compound added to the apical surface. Each compound was tested against cell cultures isolated from three or more donors. A paired two-tailed Student’s *t*-test was performed to compare the P_app_ of the compounds tested against identical isolates. The P_app_ values are reported as means.

### 4.4. MIC Determination

The minimum inhibitory concentration (MIC) assay was performed using a protocol adapted from the 2018 guidelines of the Clinical and Laboratory Standards Institute (CLSI), using the microtiter MIC method [53]. The inocula were prepared by suspending a colony from an Luria–Bertani agar plate into tryptic soy broth (TSB) and growing for 3 to 5 h at 37 °C with shaking, then adjusting the culture turbidity in TSB to an optical density at 600 nm (OD600) of 0.25 (~3 × 10^8^ cells per mL). A total of 2.5 μL (~1 × 10^6^ cells) of this suspension was added to 100 μL wells each containing compound diluted in cation-adjusted Mueller–Hinton II broth and incubated with no shaking for 24 h at 37 °C. The MIC was defined as the lowest concentration of the compound (in micrograms per milliliter) in which bacterial growth in the well was not measurable by determining its turbidity (OD600) on a BioTek Synergy 2 plate reader (BioTek Instruments Inc., Winooski, VT, USA). The reported values are the means of two replicates. The MICs were determined for *Burkholderia pseudomallei*, *Pseudomonas aeruginosa*, *Escherichia coli*, and *Staphylococcus aureus* Newman.

## 5. Conclusions

Modifying antibiotics to increase their retention in the lungs following pulmonary delivery could improve the therapeutic potential for pulmonary infections by promoting higher local concentrations and limiting systemic exposure that could lead to toxicity. Several ceftazidime analogs exhibited slower transport across a cell culture model of the lung epithelium but suffered from significantly reduced antibacterial potency. In addition, the reduction in the apparent permeability was limited and most likely driven by a slower rate of diffusion for hydrophilic derivatives of ceftazidime. These data suggest that achieving substantial increases in lung persistence for membrane impermeable antibiotics may be difficult with a molecular approach unless (1) suitable motifs are identified that promote binding to components in the lung and minimally interfere with the conjugated antibacterial’s mechanism of action or (2) high-molecular-weight prodrugs of antibacterials can outperform or work synergistically with slow-release particle formulation approaches. Future efforts are needed to identify structural motifs and evaluate antibiotic scaffolds that are better suited to retain antimicrobial potency while imbuing desirable properties to enhance pulmonary retention.

## Figures and Tables

**Table 1:** Ceftazidime analog molecular characteristics, P_app_, and MIC values.

Compound	R Group	MW (g/mol)	Predicted LogP ^1^	P_app_^2^	MIC (μg/mL) ^3^			
					Bp	Pa	Ec	Sa
Ceftazidime pentahydrate	-	636.7	−0.47	3.36 ± 0.18 2.54 ± 0.95	3	6	0.4	13
**1**	N-butane	601.7	0.27	1.91 ± 0.45	200	200	100	6
**2**	N-heptane	643.8	1.42	3.9 ± 1.1	>200	>200	100	3
**3**	Phenyl ethyl	649.7	0.82	4.65 ± 0.86	200	200	100	3
**4**	2-Chlorodopamine	716.2	0.83	2.94 ± 0.63	1.6	19	8	38
**5**	Phenyl propyl sulfone	727.8	0.43	2.71 ± 0.93	150	200	13	13
**6**	6-(4-Phenylbutoxy) hexane	778.0	2.72	2.45 ± 0.21	>200	200	100	3
**7**	mPEG3	691.8	−0.86	3.6 ± 1.1	>200	>200	100	50
**8**	mPEG5	779.9	−1.12	2.23 ± 0.22	>200	>200	100	100
**9**	mPEG8	912.0	−1.52	1.86 ± 0.39	>200	>200	>200	200
**10**	Glucosamine	707.7	−2.32	2.75 ± 0.68	38	50	3	150
**11**	Diethyl 1,2-diamine	644.8	−0.08	3.58 ± 0.50	75	150	63	25
**12**	1-Ethyl-4-methylpiperizine	671.2	−0.80	2.60 ± 0.94	>200	>200	>200	>200
**13**	Glutamic acid	675.1	−1.04	2.79 ± 0.91	200	200	13	200
**14**	Ethyl-pyrimidin-2-amine	681.2	−0.51	2.03 ± 0.60	>200	100	>200	>200
**15**	PEG5-pyrimidin-2-amine	902.0	−1.09	1.75 ± 0.06	200	>200	38	50
**16**	4-Propylthiophenyl boronic acid	739.6	0.81	4.9 ± 1.4	200	>200	50	3
**17**	4-Propylsulfonylphenyl boronic acid	771.6	−0.14	3.33 ± 0.36	200	200	50	13
**18**	4-Ethylcarbamoylphenyl boronic acid	736.6	−0.43	2.21 ± 0.45	50	50	5	25

^1^ Predicted LogP values averaged from MLogP, ALogP, ALogPs, and XLogP2 values. ^2^ Apparent permeability in units of 10^−6^ cm/s with the standard deviation reported as the error. For Ceftazidime pentahydrate, the top value is for compound set 1, and the bottom value for compound set 2. ^3^ Bp—*Burkholderia pseudomallei*; Pa—*Pseudomonas aeruginosa*; Ec—*Escherichia coli*; Sa—*Staphylococcus aureus*.

## Data Availability

The original contributions presented in this study are included in the article/Supplementary Materials. Further inquiries can be directed to the corresponding author.

## Supplementary Materials

The following supporting information can be downloaded at: https://www.mdpi.com/article/10.3390/antibiotics14020177/s1, Figure S1: Cartoon representation of transport assay; Figure S2: Plots of cumulative transport as a function of time for each ceftazidime analog; Figure S3: Correlation of Papp as a function of eight molecular descriptors; Table S1: TEER values for HBEC culture compound set 1; Table S2: TEER values for HBEC culture compound set 2; Table S3: Raw data for P_app_ comparison of ceftazidime in compound set 1 and compound set 2; Table S4: Two-tailed paired *t*-test *p* values from P_app_ comparison with ceftazidime compound set 1; Table S5: Two-tailed paired *t*-test *p* values from P_app_ comparison with ceftazidime compound set 2; Ceftazidime analog synthesis methods, characterization methods, and characterization data.
