# Supplementary material for: Impact of Hydrophobic, Hydrophilic, and Mucus-Binding Motifs on the Therapeutic Potential of Ceftazidime Analogs for Pulmonary Administration
Source: Antibiotics (Basel). 2025 Feb 11;14(2):177. doi: 10.3390/antibiotics14020177 (PMC11852049; doi:10.3390/antibiotics14020177)

Impact of Hydrophobic, Hydrophilic, and Mucus-Binding Motifs on the Therapeutic Potential of Ceftazidime Analogs for Pulmonary Administration

**Kyle D. Apley [1], Stephanie N. Johnson [1], Jian Qian [1], Indeewara Munasinghe [2], Jennifer R. Klaus [3], Srilaxmi M. Patel [2], Kathryn E. Woods [3], Samalee Banerjee [3], Josephine R. Chandler [3], Chamani Perera [2], Nathalie Baumlin [4],
Matthias Salathe [4] and Cory J. Berkland [1,5,6,*]**

1 Department of Pharmaceutical Chemistry, University of Kansas, Lawrence, KS 66045, USA; kylea@wustl.edu (K.D.A.); stephanie_clark_2010@hotmail.com (S.N.J.); jianqian1@gmail.com (J.Q.)
2 Synthetic Chemical Biology Core Laboratory, University of Kansas, Lawrence, KS 66045, USA;
aruna_munasinghe@ku.edu (I.M.); srilaxmi.malipatel@ku.edu (S.M.P.); chamani@ku.edu (C.P.)
3 Department of Molecular Biosciences, University of Kansas, Lawrence, KS 66045, USA; jennpfannklaus@gmail.com (J.R.K.); katewoods456@gmail.com (K.E.W.); samalee.banerjee@ku.edu (S.B.); jrchandler@ku.edu (J.R.C.)
4 Department of Internal Medicine, University of Kansas Medical Center, Kansas City, KS 66160, USA; nbaumlin@kumc.edu (N.B.); msalathe@kumc.edu (M.S.)
5 Department of Chemical and Petroleum Engineering, University of Kansas, Lawrence, KS 66045, USA
6 Bioengineering Graduate Program, University of Kansas, Lawrence, KS 66045, USA
* Correspondence: coryb@wustl.edu; Tel.: +1-(785)-764-0125

Table of Contents:

**Table of Contents:**	**Page #**
Table S1	3
Table S2	3
Table S3	4
Table S4	4
Table S5	5
Figure S1	6
Figure S2	7
Figure S3	8
Synthesis of Ceftazidime Analogues	9-28

Table S1: TEER values for HBEC culture compound set 1

	Lung Donor ID		
**Compound**	**N715**	**N716**	**N720**
Ceftazidime	740	590	760
1	690	760	670
8	840	630	880
9	950	700	950
11	950	630	940
15	870	720	930
**Min**	690	590	670
**Max**	950	760	950
**Mean**	840	672	855
**SD**	98	59	105

Table S2: TEER values for HBEC cultures (compound set 2)

	Lung Donor ID			
**Compound**	**L2**	**L8**	**KC29**	**L224**
Ceftazidime	1008	318	812	477
2	1066	491	766	515
3	1033	379	1000	657
4	935	403	674	479
5	1070	337	804	614
6	676	372	464	448
7	950	306	1197	578
10	919	313	858	388
12	1194	495	1002	585
13	818	245	672	864
14	1076	429	734	451
16	933	410	775	691
17	1019	486	857	502
18	1019	255	795	421
**Min**	676	245	464	388
**Max**	1194	495	1197	864
**Mean**	980	374	815	548
**SD**	121	80	168	123

Table S3: Raw data for P_{app} comparison of ceftazidime in compound set 1 and compound set 2

Compound Set	Lung Donor ID	P_{app} x10^{-6}	P value*
1	N715	3.17	0.38
	N716	3.37	
	N720	3.54	
2	L2	2.13	
	KC29	1.86	
	L224	3.63	

*To determine if P_{app} values for ceftazidime were statistically different amongst the two compounds sets, a P value was calculated using a two-tailed paired t-test. Three donor tissue cultures were used per set (n=3), and a P_{app} was calculated for each culture. A cutoff of $p<0.05$ was set as a threshold for statistical significance.

Table S4: Two-tailed paired t-test P values from P_{app} comparison with ceftazidime compound set 1

Compound	Mean P_{app} x10^{-6}	P value*
Ceftazidime	3.36	-
1	1.91	0.013
8	2.23	0.13
9	1.86	0.012
11	3.58	0.47
15	1.75	0.0073

*To determine if P_{app} values for ceftazidime analogs were statistically different from ceftazidime, P values were calculated using a two-tailed paired t-test. Three donor tissue cultures were used per ceftazidime (n=3), and a P_{app} was calculated for each culture. A cutoff of $p<0.05$ was set as a threshold for statistical significance.

suitable motif and antibiotic scaffold partners are identified that exhibit binding to components of the lung withought severely hampering antimicrobial activity

Table S5: Two-tailed paired t-test P values from P_{app} comparison with ceftazidime compound set 2

Compound	Mean P_{app} x10^{-6}	P value*
Ceftazidime	2.54	-
2	3.89	0.041
3	4.65	0.008
4	2.94	0.17
5	2.71	0.51
6	2.45	0.91
7	3.62	0.22
10	2.75	0.45
12	2.60	0.92
13	2.79	0.010
14	2.03	0.45
16	4.86	0.013
17	3.33	0.16
18	2.21	0.44

*To determine if P_{app} values for ceftazidime analogs were statistically different from ceftazidime, P values were calculated using a two-tailed paired t-test. Three donor tissue cultures were used per ceftazidime (n=3), and a P_{app} was calculated for each culture. A cutoff of $p<0.05$ was set as a threshold for statistical significance.

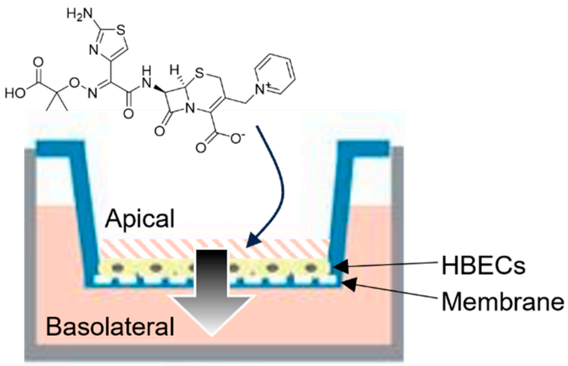

Figure S1: Cartoon representation of the human bronchial epithelial cell transwell air-liquid interface culture for the transport assay.

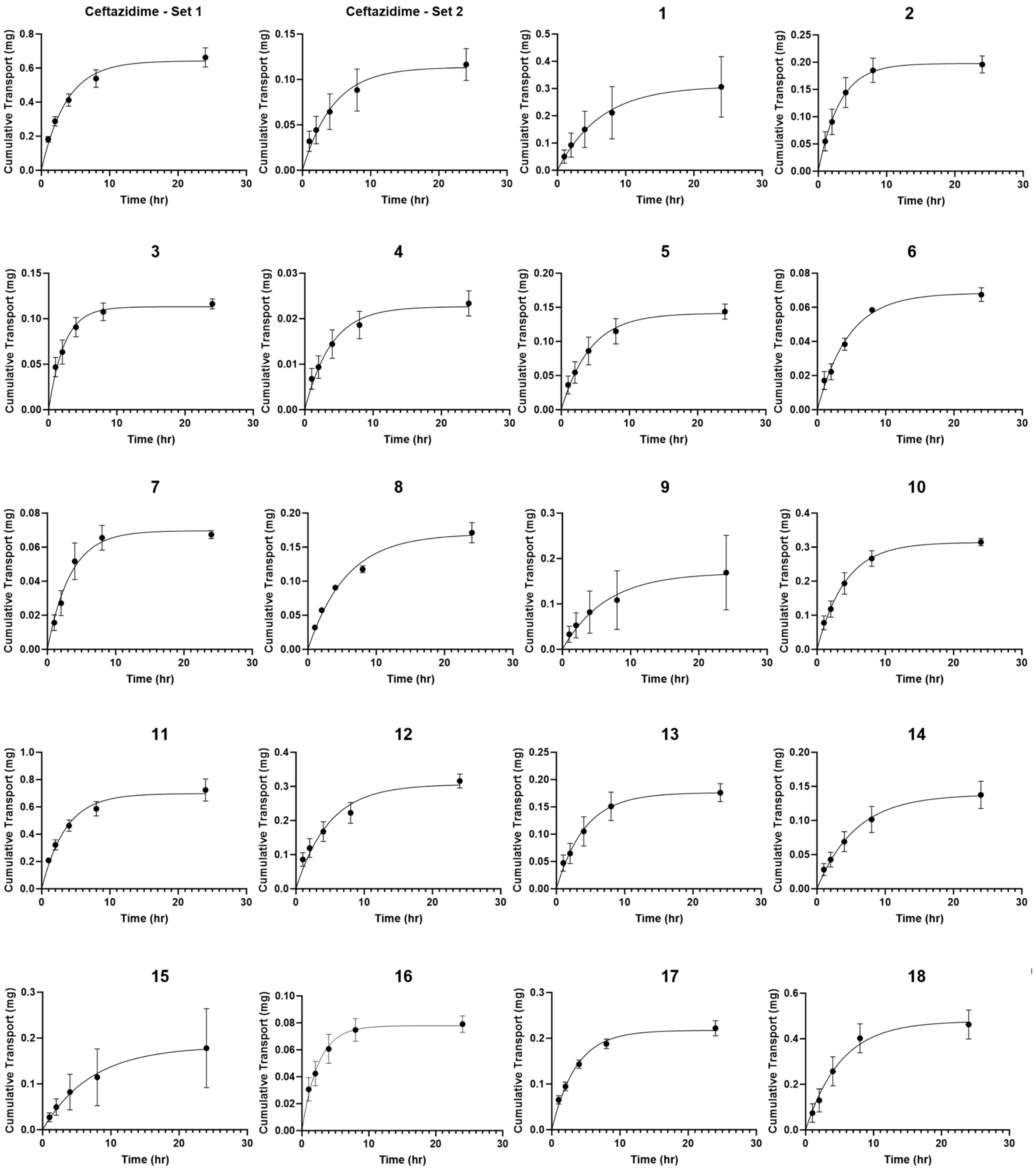

Figure S2: Plots of cumulative transport for each ceftazidime analogue as a function of time. The release kinetics are fit to a non-linear regression of the form $y=a(1-e^{-kt})$. Error bars give standard deviation.

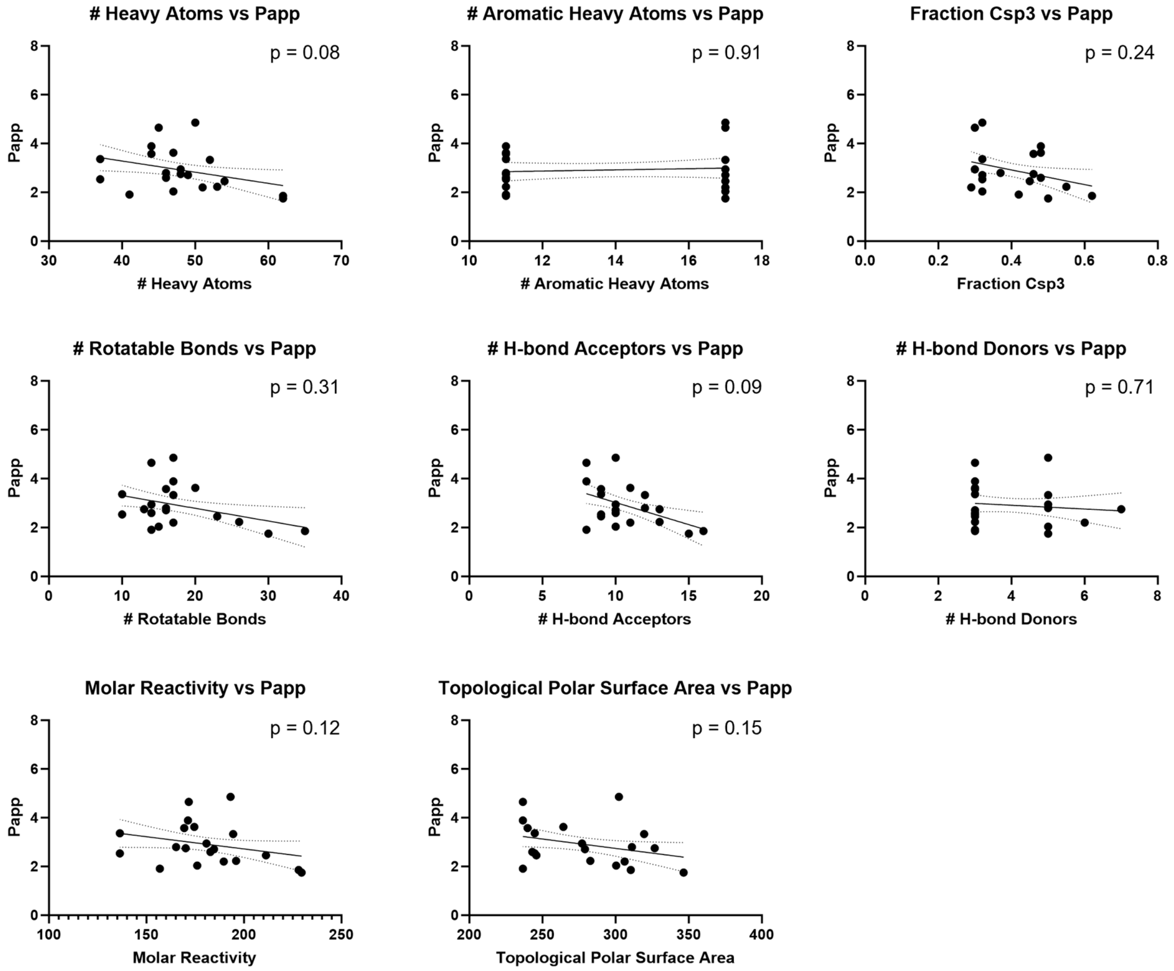

Figure S3: Plots of ceftazidime analog Papp as a function of eight additional molecular descriptors. The line depicts the linear regression representing the correlation between the respective factor and Papp with the dotted line as bounds of the 95% CI for the correlation. The p-value for the correlation is displayed over each plot. The molecular descriptor values were obtained using SwissADME.

Materials

Ceftazidime pentahydrate was purchased from Acros Organics. Respective amines were purchased from Alfa Aesar, TCI America and BroadPharm.

Analytical Characterization

Electrospray Ionization spectra in positive mode were acquired on a LCT Premier (Waters Corp., Milford MA) time of flight mass spectrometer. The instrument was operated at 10,000 resolution (W mode) with dynamic range enhancement that attenuates large intensity signals. The cone voltage was 60 eV. Spectra were acquired by accumulating data for 2 seconds per cycle. Mass correction for exact mass determinations were made automatically with the lock mass feature in the MassLynx data system. A reference compound in an auxiliary sprayer is sampled every third cycle by toggling a "shutter" between the analysis and reference needles. The reference mass is used for a linear mass correction of the analytical cycles. Samples are presented in acetonitrile as a 100 µL loop injection using an auto injector (LC PAL, CTC Analytics AG, Zwingen, Switzerland).

NMR spectra were acquired on a 500 MHz Bruker AVIII spectrometer equipped with a cryogenically-cooled carbon observe probe and B-ACs autosampler and all the samples were dissolved in 500 µL DMSO-d_6. NMR data processing was carried out using MestReNova Version: 11.0.2-18153.

Analytical HPLC traces were acquired using an Agilent 1100 quaternary pump and a Hamilton PRP-1 (polystyrene-divinylbenzene) reverse phase analytical column (7 µm particle size, 4 mm x 25 cm) with UV detection at 210 nm. The elution was achieved with gradients of water/ acetonitrile (90:10 to 0:100 containing 0.1% TFA) over 20 min.

The quantity of ceftazidime analog in transport assay samples was determined by RP-HPLC-UV. 200 μL of each sample was added into 400 μL acetonitrile, and these mixtures were refrigerated overnight. Then these mixtures were centrifuged at 14,000 xg for 5 min, and the supernatants were collected for HPLC analysis. Analytical HPLC traces were acquired using a Waters 2796 Bioseparation Module and a reverse phase Higgins Analytical PROTO 200 C18 column (5 μm particle size, 4 mm x 25 cm) with UV detection at 215 nm and 254 nm. Elution was achieved with gradients of water/ acetonitrile (95: 5 to 0: 100 containing 0.1% TFA) over 30 min.

Synthesis of Ceftazidime Analogs

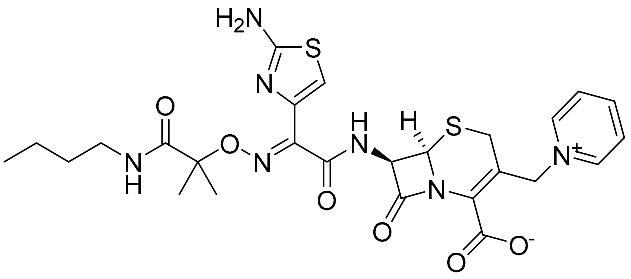

(6R,7R)-7-((E)-2-(2-aminothiazol-4-yl)-2-(((1-(butylamino)-2-methyl-1-oxopropan-2-yl)oxy)imino)acetamido)-8-oxo-3-(pyridin-1-ium-1-ylmethyl)-5-thia-1-azabicyclo[4.2.0]oct-2-ene-2-carboxylate (1). A solution of ceftazidime pentahydrate (200 mg, 0.314 mmol, 1 eq.) was prepared in dry DMF (16 mL) containing dry DIPEA (190 μL, 1.10 mmol, 3.5 eq.) and 4Å molecular sieves in a 50 mL round bottomed flask. Solid HATU (142.4 mg, 0.471 mmol, 1.2 eq.) was slowly added to the above solution and the container was then sealed and purged with Ar. The solution was stirred for 1 h under an Ar atmosphere. The solution color changed from light yellow to dark red with no precipitation. Butylamine (29.9 mg, 0.408 mmol, 1.3 eq.) was then injected, followed by injection of dry DIPEA (190 μL, 1.10 mmol, 3.5 eq.). The reaction solution was stirred

for 15 h at room temperature under Ar. Purification of 1 was carried out by reverse phase HPLC using a prep C18 5µm column (Column ID: 19 mm x 150 mm) with a t_R = 11.2 min. The solvent profile used in purification is as follows: t=0 to 3.5 min-isocratic gradient of 10% MeCN: 90% water, t=3.5 to 29.5 min-MeCN increased from 10% to 100%. Compound **1** was collected and lyophilized to give a 21.2 mg light-yellow solid (10 % yield). ^{1}H NMR (500 MHz, DMSO-d6) 9.75 (1H, d, J = 8.1 Hz,), 9.05 (2H, d, J = 5.7 Hz), 8.67 (1H, t, J = 7.8 Hz), 8.22 (2H, t, J = 6.7 Hz), 7.34 (2H, broad s), 7.10 (1H, t, J = 5.6 Hz), 6.80 (1H, s), 5.98 (1H, dd, J = 4.92 Hz, 8.2 Hz), 5.63 (1H, d, J = 14.6 Hz), 5.54 (1H, d, J = 14.7 Hz) 5.24 (1H, d, J = 4.9 Hz, CH), 3.56 (1H, dd, J = 18.8 Hz), 3.41 (1H, dd, J = 18.3 Hz), 3.06 (2H, m, CH2), 1.40 (3H, s), 1.39 (3H, s), 1.34 (2H, m), 1.21 (2H, m), 0.78 (3H, t, 7.3 Hz). ^{13}C NMR (500 MHz, DMSO-d6) 173.5, 169.6, 164.4, 163.4, 163.0, 149.0, 146.8, 145.6 (3 overlapping C), 129.6, 128.9 (2 overlapping C), 120.5, 110.5, 84.1, 61.1, 59.4, 58.0, 38.7, 31.6, 25.9, 25.0, 24.5, 19.8, 14.1. HRMS (ESI$^+$) calculated for $C_{26}H_{31}N_7O_6S_2$ ([M+H]$^+$) 602.1850; found 602.1982. Retention time 7.20 mins. Analogous procedures were followed for the remaining ceftazidime derivatives by using the corresponding amine.

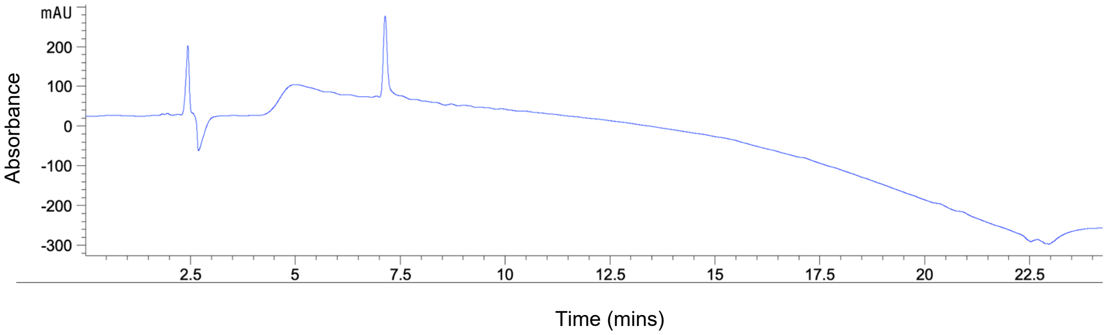

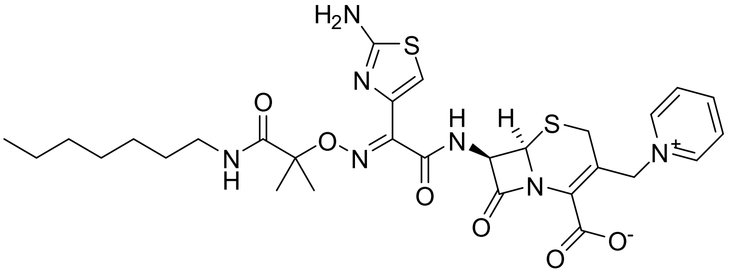

(6R,7R)-7-((E)-2-(2-aminothiazol-4-yl)-2-(((1-(heptylamino)-2-methyl-1-oxopropan-2-yl)oxy)imino)acetamido)-8-oxo-3-(pyridin-1-ium-1-ylmethyl)-5-thia-1-azabicyclo[4.2.0]oct-2-ene-2-carboxylate (2). Prepared in an analogous manner to compound **1**. A reaction on a 0.31 mmol (200 mg) scale yielded 11.3 mg of compound **2** as a light-orange solid (6 % yield). ^{1}H NMR (500 MHz, DMSO-d6), 9.73 (1H, d, J = 8.1 Hz), 9.05 (2H, d, J = 5.5 Hz), 8.66 (1H, t, J = 7.7 Hz), 8.20 (2H, m, J = 6.9 Hz), 7.30 (2H, s, NH2), 7.11 (1H, t, J = 5.9 Hz), 6.78 (1H, s, CH), 5.95 (1H, dd, J = 4.9 Hz, 8.02 Hz), 5.61 (1H, d, J = 14.6 Hz), 5.52 (1H, d, J = 14.6 Hz), 5.24 (1H, d, J = 5.1 Hz, CH), 3.54 (1H, d, J = 18.5 Hz), 3.41 (1H, d, J = 17.9 Hz), 3.04 (2H, m), 1.38 (3H, s), 1.37 (3H, s), 1.20 (10H, m), 0.83 (3H, t, J = 7.01 Hz). ^{13}C NMR (500 MHz, DMSO-d6) 173.2, 168.6, 164.00, 163.3, 162.9, 149.9, 146.3, 145.0 (2 overlapping C), 144.8, 142.4, 128.4 (2 overlapping C), 119.94, 109.6, 83.24, 60.7, 58.9, 57.6, 38.6, 31.2, 29.1, 28.5, 26.2, 24.6, 24.5, 24.2, 22.1, 14.0. HRMS (ESI^{+}) calculated for $C_{29}H_{37}N_7O_6S_2$ ([M+H]$^{+}$) 644.2320, ([M+2H]$^{2+}$) 322.6197; found 644.2406, 322.6193. Retention time 9.25 mins.

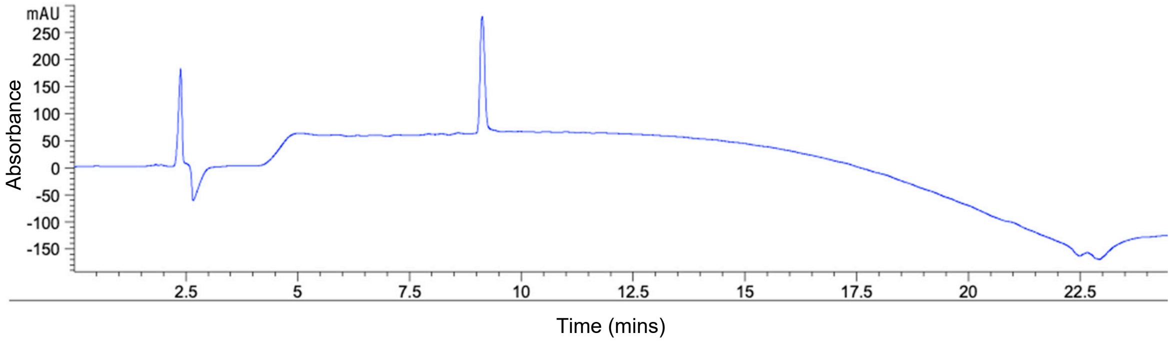

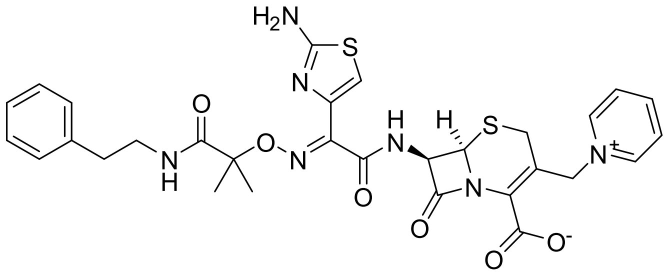

(6R,7R)-7-((E)-2-(2-aminothiazol-4-yl)-2-(((2-methyl-1-oxo-1-(phenethylamino)propan-2-yl)oxy)imino)acetamido)-8-oxo-3-(pyridin-1-ium-1-ylmethyl)-5-thia-1-azabicyclo[4.2.0]oct-2-ene-2-carboxylate (3). Prepared in an analogous manner to compound **1**. A reaction on a 0.31 mmol (200 mg) scale yielded 13.2 mg of compound **3** as a light-yellow solid (6.5% yield). ^{1}H NMR (500 MHz, DMSO-d6) 9.71 (1H, d, J = 8.0 Hz), 9.03 (2H, d, J = 5.8 Hz), 8.62 (1H, t, J = 7.6 Hz), 8.18 (2H, t, J = 6.9 Hz), 7.32 (2H, broad s), 7.30-7.12 (6H, m), 6.79 (1H, s), 5.97 (1H, dd, J = 4.75 Hz, 8.13 Hz), 5.61 (1H, d, J = 14.8 Hz), 5.52 (1H, d, J = 14.7 Hz) 5.24 (1H, d, J = 5.08 Hz), 3.56 (1H, d, J = 18.3 Hz), 3.41 (1H, d, 18.2 Hz), 3.26 (2H, m), 1.35 (6H, s), 1.23 (2H, m). ^{13}C NMR (500 MHz, DMSO-d6) 173.3, 168.7, 164.2, 162.9, 162.3, 146.3, 145.0 (3 overlapping C), 144.8, 139.3, 129.2, 128.7 (4 overlapping C), 128.4 (2 overlapping C), 126.0, 120.0, 109.9, 83.3, 60.7, 59.0, 57.7, 35.8, 35.2, 25.5, 24.6, 24.1. HRMS (ESI$^+$) calculated for $C_{30}H_{31}N_7O_6S_2$ ([M+H]$^+$) 650.1850; found 650.1830. Retention time 7.97 mins.

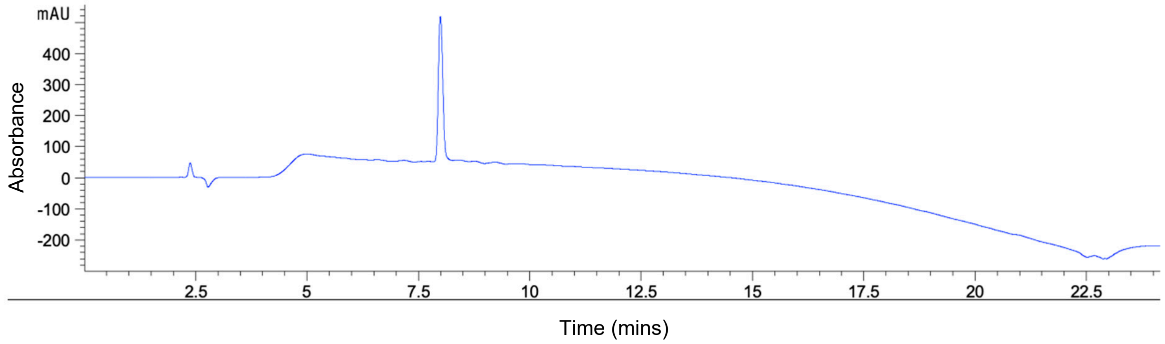

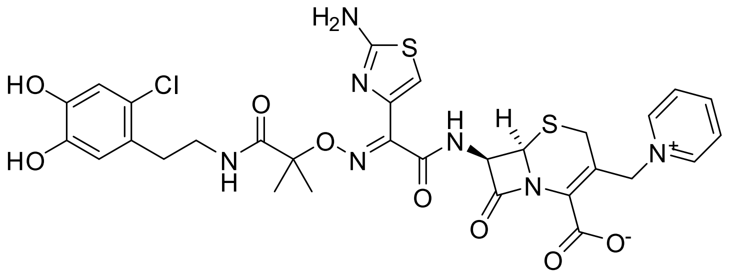

(6R,7R)-7-((E)-2-(2-aminothiazol-4-yl)-2-(((1-((2-chloro-4,5-dihydroxyphenethyl)amino)-2-methyl-1-oxopropan-2-yl)oxy)imino)acetamido)-8-oxo-3-(pyridin-1-ium-1-ylmethyl)-5-thia-1-azabicyclo[4.2.0]oct-2-ene-2-carboxylate (4). Prepared in an analogous manner to compound 1. A reaction on a 0.31 mmol (200 mg) scale yielded 13 mg of compound **4** as a white powder (6% yield). ^{1}H NMR (500 MHz, DMSO-d6) 9.72 (1H, d, J = 8.2 Hz), 9.04 (2H, d, J = 5.6 Hz), 8.61 (1H, t, J = 7.6 Hz), 8.17 (2H, t, J = 7.1 Hz), 7.41 (2H, broad s), 7.27 (1H, t, J= 5.9 Hz), 6.80 (1H, s), 6.70 (1H, s), 6.60 (1H, s), 5.98 (1H, dd, J = 4.9 Hz, 8.3 Hz), 5.61 (2H, dd, J = 14.4 Hz, 37.0 Hz), 5.25 (1H, d, J = 5.0 Hz), 3.48 (2H, dd, J = 17.5 Hz, 71.0 Hz), 3.20 (2H, m), 2.60 (2H, m) 1.38 (6H, s). ^{13}C NMR (500 MHz, DMSO-d6) 173.8, 169.3, 164.7, 163.4 (2 overlapping C), 149.8, 146.7, 145.4 (2 overlapping C), 145.2 144.9, 142.2, 129.7, 128.8 (2 overlapping C), 127.1, 122.1, 120.5, 118.1 (2 overlapping C), 116.3, 110.3, 83.9, 61.1, 59.2, 58.1, 32.8, 25.9, 25.3, 24.5 HRMS (ESI+) calculated for $C_{30}H_{30}ClN_7O_8S_2$ ($[M+H]^+$) 716.1364; found 716.1264. Retention time 7.25 mins.

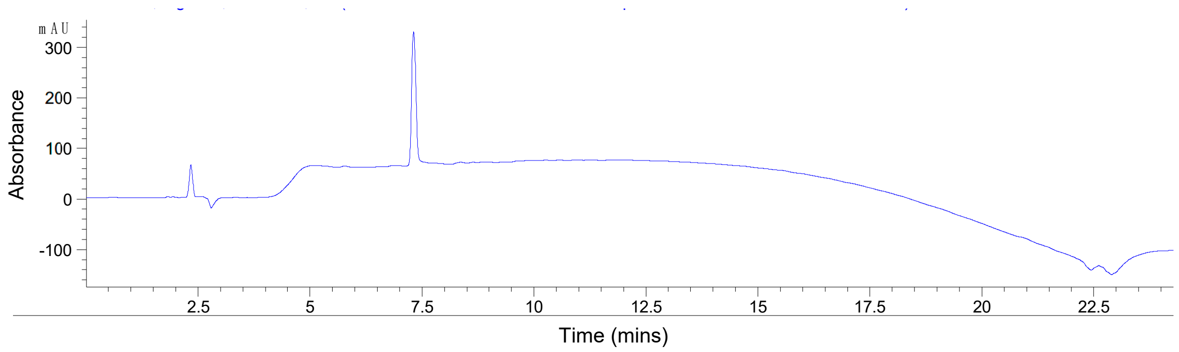

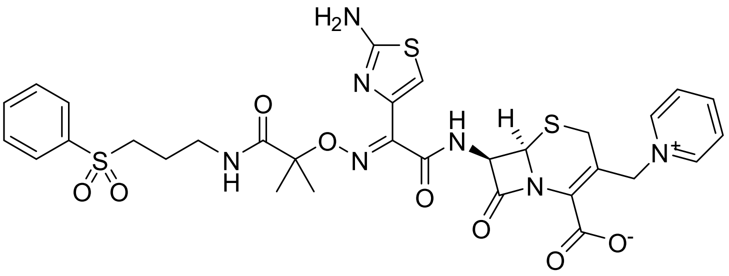

(6R,7R)-7-((E)-2-(2-aminothiazol-4-yl)-2-(((2-methyl-1-oxo-1-((3-(phenylsulfonyl)propyl)amino)propan-2-yl)oxy)imino)acetamido)-8-oxo-3-(pyridin-1-ium-1-ylmethyl)-5-thia-1-azabicyclo[4.2.0]oct-2-ene-2-carboxylate (5). Prepared in an analogous manner to compound 1. A reaction on a 0.31 mmol (200 mg) scale yielded 25 mg of compound **5** as a yellow solid (11.07 % yield). ^{1}H NMR (500 MHz, DMSO-d6) δ 9.71 (1H, d, *J* = 8.3 Hz), 9.07 – 9.02 (2H, m), 8.67 – 8.60 (1H, m), 8.19 (2H, dd, *J* = 7.8, 6.5 Hz), 7.82 (2H, dd, *J* = 8.4, 1.3 Hz), 7.78 – 7.72 (1H, m), 7.65 (2H, dd, *J* = 8.3, 7.1 Hz), 6.82 (1H, s), 6.01 (1H, dd, *J* = 8.4, 5.0 Hz), 5.66 – 5.48 (2H, m), 5.27 (1H, d, *J* = 4.9 Hz), 3.59 (1H, d, *J* = 18.4 Hz), 3.45 (1H, d, *J* = 18.3 Hz), 3.23 (2H, dd, J = 9.9, 6.1 Hz), 3.19 – 3.12 (1H, m), 1.64 (2H, dd, *J* = 11.5, 6.7 Hz), 1.36 (6H, d, J = 2.0 Hz).^{13}C NMR (126 MHz, DMSO-d6) δ 174.1, 169.2, 164.8, 163.5, 163.4, 158.2, 150.2, 146.7, 145.5 (2 overlapping C), 142.6, 139.2, 134.3, 129.9, 128.9 (2 overlapping C), 128.0, 120.7, 110.3, 109.8, 107.9, 105.9, 100.0, 83.7, 61.1, 59.3, 52.9, 37.4, 25.4, 24.3, 23.2. HRMS (ESI^{+}) calculated for $C_{31}H_{33}N_{7}O_{8}S_{3}$ ([M+H]$^{+}$) 728.1625; found 728.15922. Retention time 7.58 mins.

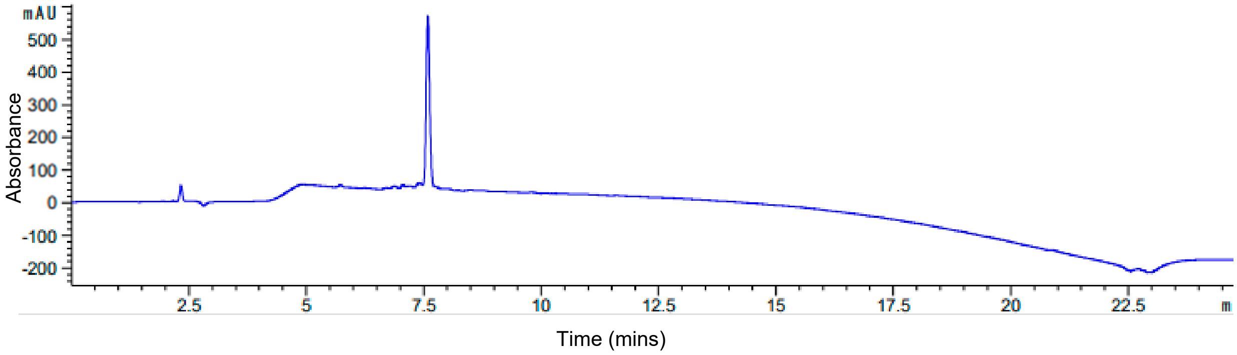

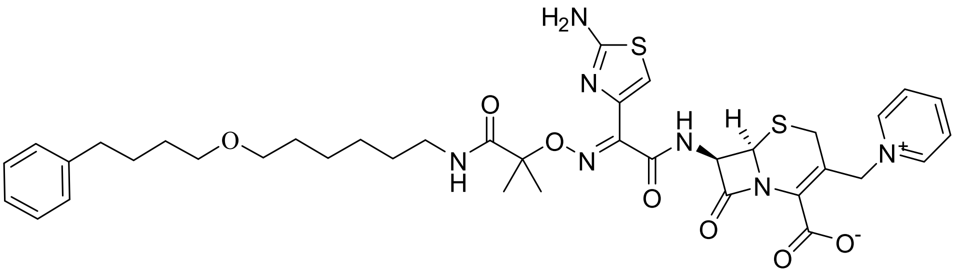

(6R,7R)-7-((E)-2-(2-aminothiazol-4-yl)-5,5-dimethyl-6-oxo-18-phenyl-4,14-dioxa-3,7-diazaoctadec-2-enamido)-8-oxo-3-(pyridin-1-ium-1-ylmethyl)-5-thia-1-azabicyclo[4.2.0]oct-2-ene-2-carboxylate (6). Prepared in an analogous manner to compound 1. A reaction on a 0.31 mmol (200 mg) scale yielded 32 mg of compound **6** as a light yellow solid (13.1 % yield). ^{1}H NMR (500 MHz, DMSO-d6) 9.74 (1H, d, J = 8.0 Hz), 9.01 (2H, d, J = 6.1 Hz), 8.65 (1H, t, J = 7.9 Hz), 8.18 (2H, t, J = 7.1 Hz), 7.35 (2H, broad s), 7.27-7.10 (6H, m), 6.78 (1H, s), 5.95 (1H, dd, J = 5.0 Hz, 8.0 Hz), 5.57 (2H, dd, J =14.7 Hz, 37.4 Hz), 5.24 (1H, d, J = 5.1 Hz), 3.54 (2H, dd, J =17.8 Hz, 69.3 Hz), 3.32 (2H, t, 6.3 Hz), 3.26 (2H, t, J = 6.4 Hz), 3.04 (2H, m), 2.56 (2H, t, J = 7.6 Hz)), 1.58 (2H, m), 1.48 (2H, m), 1.40-1.30 (10H, m), 1.20 (4H, m). ^{13}C NMR (500 MHz, DMSO-d6) 173.2, 168.6, 164.0, 163.3, 162.9, 146.3, 145.0 (3 overlapping C), 144.8, 142.2, 128.4, 128.3 (4 overlapping C), 128.2 (2 overlapping C), 125.6, 119.4, 109.6, 83.24, 69.9, 69.7, 60.7, 60.1, 58.9, 38.5, 34.9, 29.1, 29.0, 28.8, 27.7, 26.0, 25.5, 24.6, 24.5, 24.2. HRMS (ESI+) calculated for $C_{38}H_{47}N_7O_7S_2$ ($[M+H]^+$) 778.3051; found 778.3065. Retention time 11.50 mins.

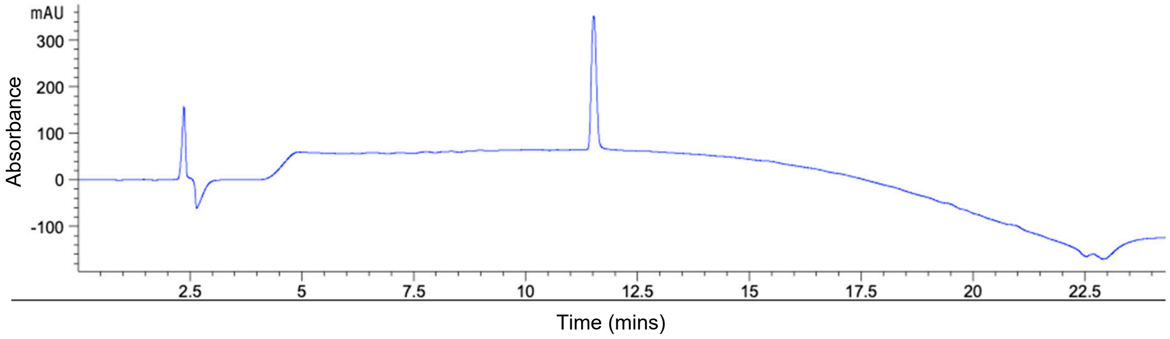

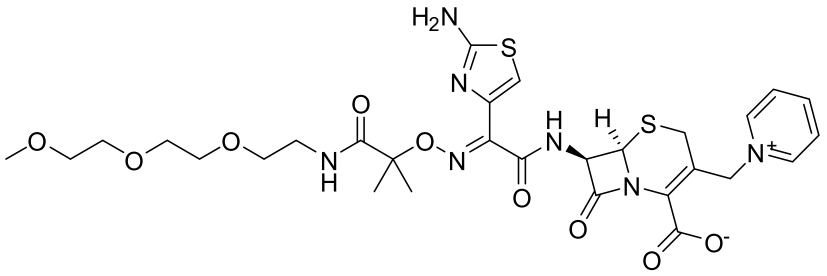

(6R,7R)-7-((E)-16-(2-aminothiazol-4-yl)-13,13-dimethyl-12-oxo-2,5,8,14-tetraoxa-11,15-diazaheptadec-15-en-17-amido)-8-oxo-3-(pyridin-1-ium-1-ylmethyl)-5-thia-1-azabicyclo[4.2.0]oct-2-ene-2-carboxylate (7). Prepared in an analogous manner to compound 1. A reaction on a 0.31 mmol (200 mg) scale yielded 10 mg of compound **7** as a white powder (5 % yield). ^{1}H NMR (500 MHz, DMSO-d6) 9.70 (1H, d, J = 8.2 Hz), 9.04 (2H, d, J = 6.0 Hz), 8.66 (1H, t, J = 7.8 Hz), 8.21 (2H, t, J = 7.0 Hz), 7.41 (2H, broad s), 7.18 (1H, t, J= 6.1 Hz), 6.80 (1H, s), 5.96 (1H, dd, J = 5.0 Hz, 8.2 Hz), 5.57 (2H, dd, J = 14.4 Hz, 39.3 Hz), 5.24 (1H, d, J = 5.0 Hz), 3.57-3.34 (12H, m) 3.22 (3H, s), 3.20 (2H, m) 1.38 (6H, s). ^{13}C NMR (500 MHz, DMSO-d6) 173.6, 168.7, 164.2 , 162.9 (2 overlapping C), 149.5, 146.3, 145.0 (2 overlapping C), 141.9, 129.3, 128.4 (2 overlapping C), 120.0, 109.9, 83.4, 71.3, 69.7 (2 overlapping C), 69.5, 68.7, 60.7, 58.9, 58.1, 57.6, 38.6, 25.0, 24.7, 24.0, HRMS (ESI^{+}) calculated for $C_{29}H_{37}N_{7}O_{9}S_{2}$ ([M+H]$^{+}$) 692.2167; found 692.2178. Retention time 6.40 mins.

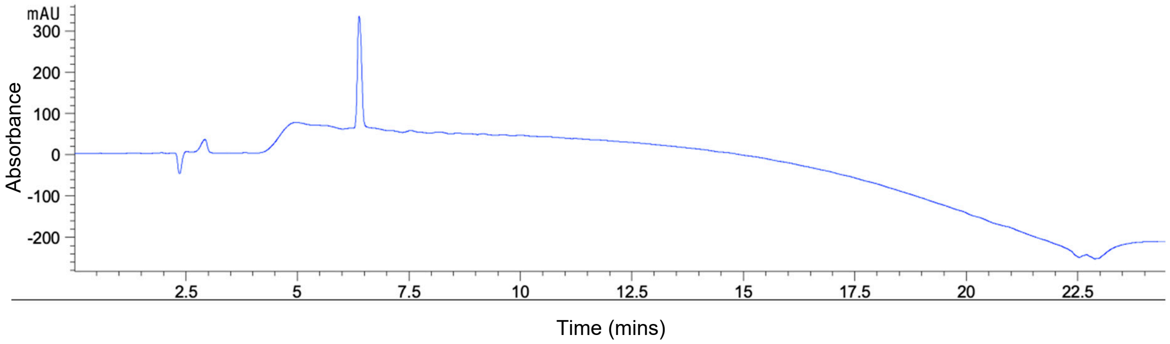

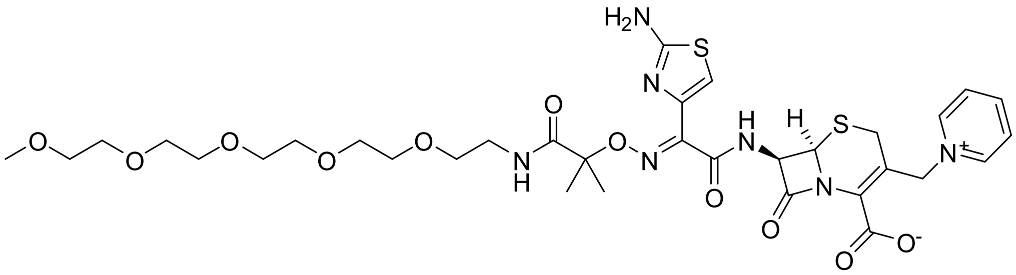

(6R,7R)-7-((E)-22-(2-aminothiazol-4-yl)-19,19-dimethyl-18-oxo-2,5,8,11,14,20-hexaoxa-17,21-diazatricos-21-en-23-amido)-8-oxo-3-(pyridin-1-ium-1-ylmethyl)-5-thia-1-azabicyclo[4.2.0]oct-2-ene-2-carboxylate (8). Prepared in an analogous manner to compound **1**. A reaction on a 0.31 mmol (200 mg) scale yielded 21.6 mg of compound **8** as a light yellow solid (8.8 % yield). ^{1}H NMR (500 MHz, DMSO-d6) 9.70 (1H, d, J = 8.0 Hz), 9.04 (2H, d, J = 6.2 Hz), 8.66 (1H, t, J = 8.1 Hz), 8.21 (2H, t, J = 6.9 Hz), 7.32 (2H, broad s), 7.17 (1H, t, J= 5.6 Hz), 6.79 (1H, s), 5.96 (1H, dd, J = 4.7 Hz, 7.8 Hz), 5.61 (1H, d, J = 14.3 Hz), 5.51 (1H, d, J = 14.8 Hz), 5.24 (1H, d, J = 5.1 Hz), 3.57-3.32 (23H, m) 3.23 (2H, m), 1.38 (6H, s).). ^{13}C NMR (500 MHz, DMSO-d6) 173.7, 168.6, 166.4, 163.0, 161.7, 149.9, 146.0, 145 (2 overlapping C), 142.5, 128.0 (3 overlapping C), 124.0, 110.0, 83.2, 71.3, 69.8 (5 overlapping C), 69.7, 69.6 (2 overlapping C), 64.3, 59.5, 58.1, 53.4, 38.6, 24.8, 24.7, 24.1. HRMS (ESI^{+}) calculated for $C_{33}H_{45}N_7O_{11}S_2$ ($[M+H]^{+}$) 780.2691; found 780.2767. Retention time 6.85 mins.

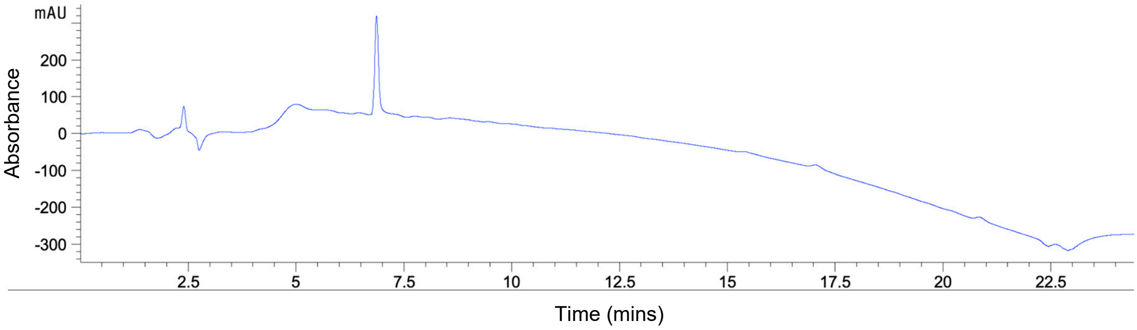

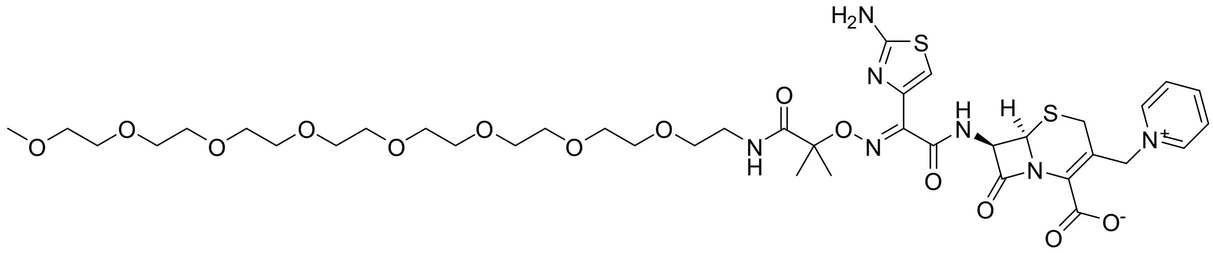

(6R,7R)-7-((E)-31-(2-aminothiazol-4-yl)-28,28-dimethyl-27-oxo-2,5,8,11,14,17,20,23,29-nonaoxa-26,30-diazadotriacont-30-en-32-amido)-8-oxo-3-(pyridin-1-ium-1-ylmethyl)-5-thia-1-azabicyclo[4.2.0]oct-2-ene-2-carboxylate (9). Prepared in an analogous manner to compound 1. A reaction on a 0.31 mmol (200 mg) scale yielded 8 mg of compound **9** as a white powder (3% yield). ^{1}H NMR (500 MHz, DMSO-d6) 9.70 (1H, d, J = 8.1 Hz), 9.04 (2H, d, J = 5.7 Hz), 8.66 (1H, t, J = 7.8 Hz), 8.21 (2H, t, J = 7.3 Hz), 7.36 (2H, broad s), 7.17 (1H, t, J= 5.9 Hz), 6.79 (1H, s), 5.97 (1H, dd, J = 4.9 Hz, 8.2 Hz), 5.56 (2H, dd, J = 14.8 Hz, 47.8 Hz), 5.24 (1H, d, J = 4.9 Hz), 3.30-3.60 (32H, m) 3.23 (3H, m), 3.20 (2H, m) 1.38 (6H, s). ^{13}C NMR (500 MHz, DMSO-d6) 173.6, 168.7, 164.2, 163.0 (2 overlapping C), 158.11, 146.3, 145.0 (2 overlapping C), 129.3, 128.4 (2 overlapping C), 120.0, 109.8, 83.3, 71.3, 69.8, 69.7 (10 overlapping carbons), 69.6, 69.5 (2 overlapping C), 68.7, 60.7, 59.0, 58.0, 57.6, 38.7, 25.4, 24.7, 24.1, HRMS (ESI^{+}) calculated for $C_{39}H_{57}N_{7}O_{14}S_{2}$ ([M+H]$^{+}$) 912.3483; found 912.3454. Retention time 7.46 mins.

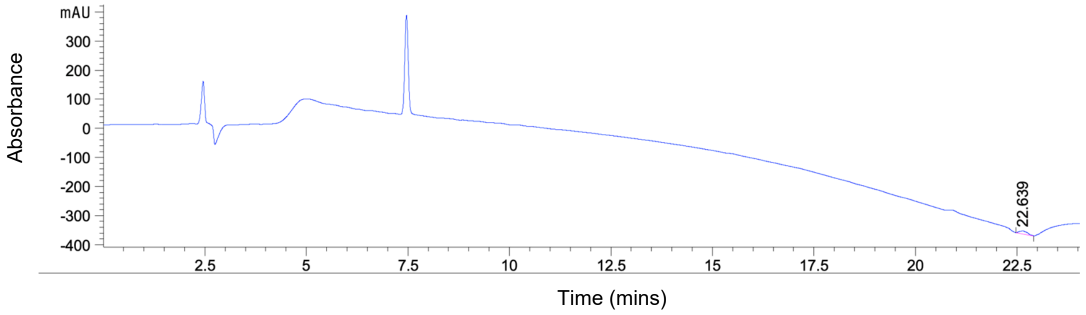

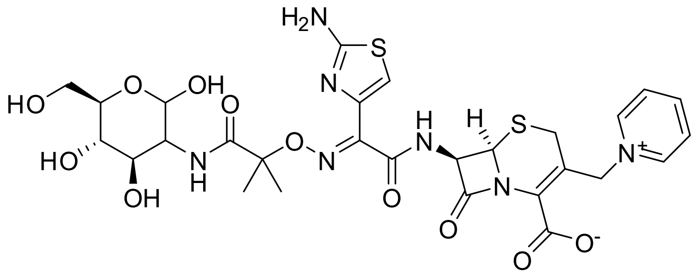

(6R,7R)-7-((E)-2-(2-aminothiazol-4-yl)-2-(((2-methyl-1-oxo-1-(((4R,5S,6R)-2,4,5-trihydroxy-6-(hydroxymethyl)tetrahydro-2H-pyran-3-yl)amino)propan-2-yl)oxy)imino)acetamido)-8-oxo-3-(pyridin-1-ium-1-ylmethyl)-5-thia-1-azabicyclo[4.2.0]oct-2-ene-2-carboxylate (10). Prepared in an analogous manner to compound **1**.A reaction on a 0.31 mmol (200 mg) scale yielded 18 mg of compound **10** as a light yellow solid (8.2 % yield). ^{1}H NMR (500 MHz, DMSO-d$_6$) δ 9.63 (1H, m), 9.60 (1H, s), 9.43 (1H, d, J = 6.1 Hz), 9.16 (1H, s), 8.59 (1H, t, J = 7.9 Hz), 8.17 (2H, t, J = 7.1 Hz), 7.24 (2H, d, J = 12.9 Hz), 6.82 (1H, d, J = 8.0 Hz), 6.54 (1H, d, J = 6.3 Hz), 5.71 (1H, s), 5.65 (1H, d, J = 13.4 Hz), 5.13 (2H, d, J = 5.0 Hz), 4.95 (1H, br. s), 4.87 (1H, br. s), 4.58 (1H, br. s), 4.06 (1H, br. s), 3.54 (2H, dd, J = 14.4, 5.7 Hz), 3.12 – 3.03 (2H, m), 1.42 (6H, d, J = 1.5 Hz).^{13}C NMR (126 MHz, DMSO-d$_6$) δ 173.9, 169.0, 163.8, 163.1, 162.4, 150.7, 150.1, 146.1, 145.6 (2 overlapping C), 142.8, 139.3, 136.6, 128.5 (2 overlapping C), 124.4, 110.3, 90.8, 83.7, 72.6, 71.2, 71.1, 61.5, 59.2, 57.9, 55.0, 25.0, 24.4. HRMS (ESI$^+$) calculated for $C_{28}H_{33}N_7O_{11}S_2$ ([M+H]$^+$) 708.1752; found 708.1669. Retention time 5.75 min.

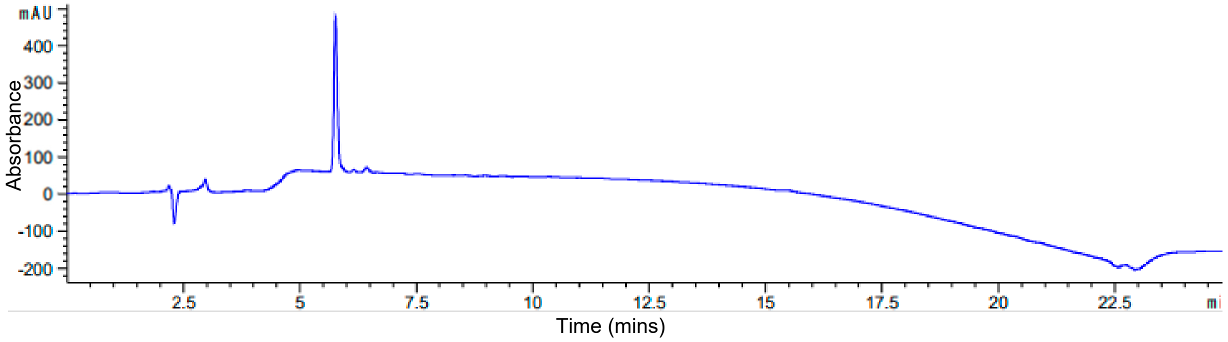

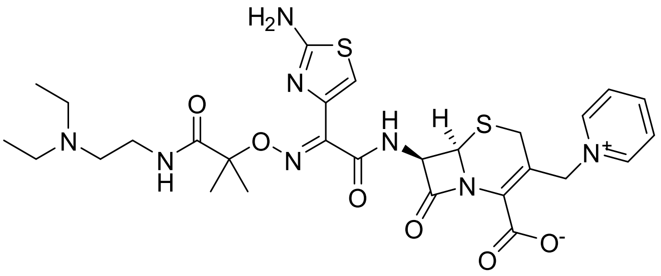

(6R,7R)-7-((E)-2-(2-aminothiazol-4-yl)-10-ethyl-5,5-dimethyl-6-oxo-4-oxa-3,7,10-triazadodec-2-enamido)-8-oxo-3-(pyridin-1-ium-1-ylmethyl)-5-thia-1-azabicyclo[4.2.0]oct-2-ene-2-carboxylate (11). Prepared in an analogous manner to compound 1. A reaction on a 0.31 mmol (200 mg) scale yielded 10 mg of compound **11** as a white powder (5% yield). ^{1}H NMR (500 MHz, DMSO-d6), 9.67 (1H, d, J = 7.8 Hz), 9.05 (2H, d, J = 6.0 Hz), 8.66 (1H, t, J = 7.7 Hz), 8.22 (2H, 2, J = 6.9 Hz), 7.58 (1H, t, J = 5.2 Hz) 7.44 (2H, broad s), 6.83 (1H, s), 5.97 (1H, dd, J = 5.0 Hz, 8.2 Hz), 5.57 (2H, dd, J = 13.8 Hz, 30.1 Hz), 5.24 (1H, d, J = 4.9 Hz, CH), 3.62-3.35 (4H, m), 3.11 (6H, m), 1.38 (6H, d, J= 3.5 Hz), 1.14 (6H, t, J = 7.2 Hz). ^{13}C NMR (500 MHz, DMSO-d6), 174.3, 168.9, 164.1, 162.9 (2 overlapping carbons), 146.3, 145.0 (2 overlapping carbons), 129.3, 128.4 (2 overlapping carbons), 119.9, 117.4, 115.1, 110.4, 83.3, 60.7, 58.9, 57.6, 49.4, 47.0 (2 overlapping carbons), 34.0, 25.5, 24.3 (2 overlapping carbons), 8.6 (2 overlapping carbons). HRMS (ESI^{+}) calculated for $C_{28}H_{36}N_8O_6S_2$ ([M+H]$^{+}$) 645.2277; found 645.2237. Retention time 5.50 mins.

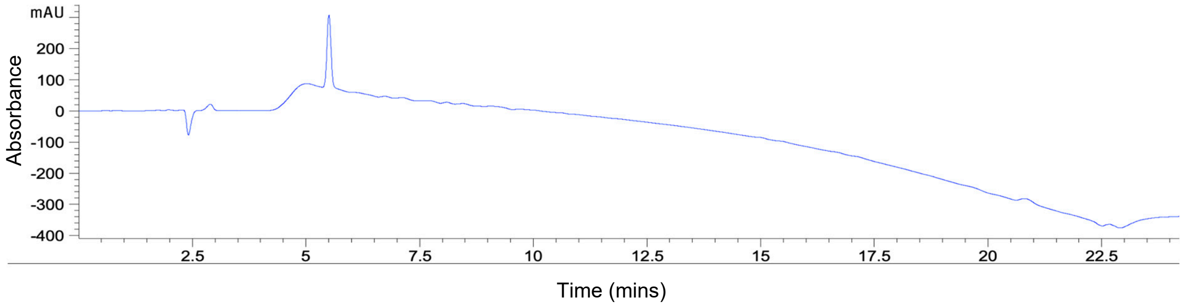

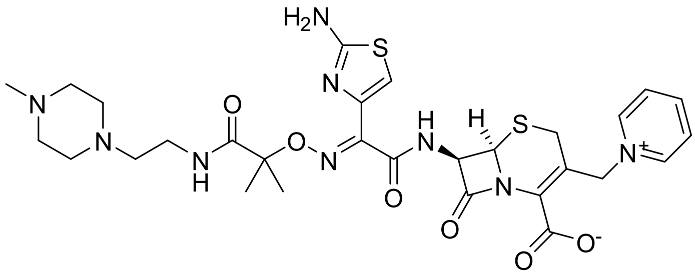

(6R,7R)-7-((E)-2-(2-aminothiazol-4-yl)-2-(((2-methyl-1-((2-(4-methylpiperazin-1-yl)ethyl)amino)-1-oxopropan-2-yl)oxy)imino)acetamido)-8-oxo-3-(pyridin-1-ium-1-ylmethyl)-5-thia-1-azabicyclo[4.2.0]oct-2-ene-2-carboxylate (12). Prepared in an analogous manner to compound 1. A reaction on a 0.31 mmol (200 mg) scale yielded 6 mg of compound **12** as a white powder (7% yield). ^{1}H NMR (500 MHz, DMSO-d6) 9.55 (1H, d, J = 7.8 Hz), 9.15 (2H, d, J = 5.4 Hz), 8.61 (1H, t, J = 7.9 Hz), 8.16 (2H, t, J = 7.2 Hz), 7.28 (2H, broad s), 7.10 (1H, t, J= 5.8 Hz), 6.83 (1H, s), 5.66 (1H, d, J = 13.8 Hz), 5.43 (1H, dd, J = 3.9 Hz, 7.7 Hz), 5.34 (2H, m), 3.22 (2H, m), 3.16 (2H, m), 2.30-2.18 (10H, m) 2.09 (3H, s), 1.40 (6H, d, J= 5.8 Hz). ^{13}C NMR (500 MHz, DMSO-d6) 174.2, 169.4, 166.7, 163.5, 161.7, 149.9, 145.9, 145.0 (2 overlapping C), 142.5, 128.0 (3 overlapping C), 123.9, 109.9, 83.2, 71.3, 69., 69.6 (3 overlapping C), 64.3, 59.5, 58.1, 53.4, 38.6, 24.8, 24.7, 24.1 HRMS (ESI^{+}) calculated for $C_{29}H_{37}N_{9}O_{6}S_{2}$ ([M+H]$^{+}$) 672.2386; found 672.2441. Retention time 3.72 mins.

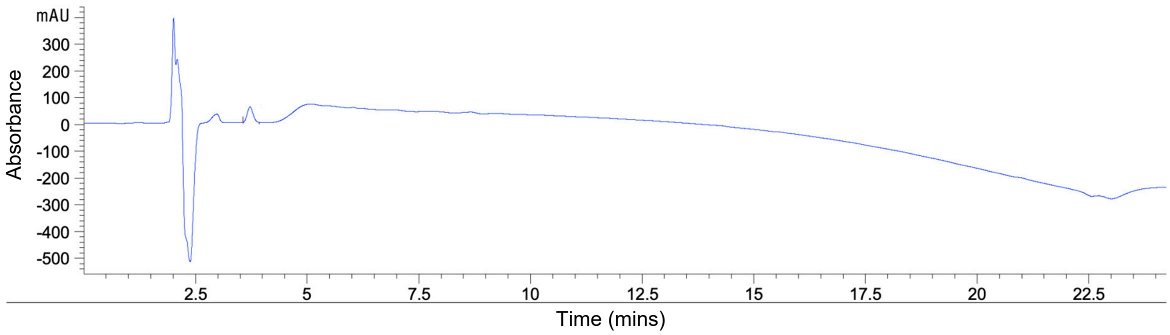

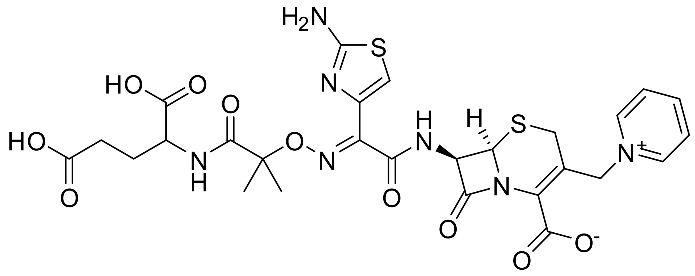

(6R,7R)-7-((E)-2-(2-aminothiazol-4-yl)-2-(((1-((1,3-dicarboxypropyl)amino)-2-methyl-1-oxopropan-2-yl)oxy)imino)acetamido)-8-oxo-3-(pyridin-1-ium-1-ylmethyl)-5-thia-1-azabicyclo[4.2.0]oct-2-ene-2-carboxylate (13). Prepared in an analogous manner to compound **1**. A reaction on a 0.31 mmol (200 mg) scale yielded 5 mg of compound **13** as a yellow solid (2 % yield). ^{1}H NMR (500 MHz, DMSO-d6) 9.70 (1H, d, J = 8.1 Hz), 9.04 (2H, d, J = 6.0 Hz), 8.65 (1H, t, J = 8.4 Hz), 8.20 (2H, t, J = 7.1 Hz), 7.28 (1H, t, J= 8.3 Hz), 7.26 (2H, broad s), 6.81 (1H, s), 5.96 (1H, dd, J = 4.9 Hz, 8.1 Hz), 5.56 (2H, dd, J = 14.6 Hz, 40.1 Hz), 5.24 (1H, d, J = 4.9 Hz), 4.22 (2H, m), 3.48 (2H, dd, 18.1 Hz, 54.6 Hz), 2.21 (2H, t, J=7.6 Hz) 1.88 (2H, m), 1.40 (6H, d, J= 5.3 Hz). ^{13}C NMR (500 MHz, DMSO-d6) 174.3, 174.0, 173.5, 169.1, 163.9, 163.4 (2 overlapping C), 150.6, 145.5 (2 overlapping C), 145.2 142.6, 128.8 (2 overlapping C), 128.6, 119.0, 116.6, 83.4, 61.4, 59.2, 58.0, 53.5, 51.5, 30.3, 26.7, 25.1, 24.3 HRMS (ESI^{+}) calculated for $C_{27}H_{29}N_7O_{10}S_2$ ([M+H]$^{+}$) 676.1495; found 676.1524. Retention time 5.50 mins.

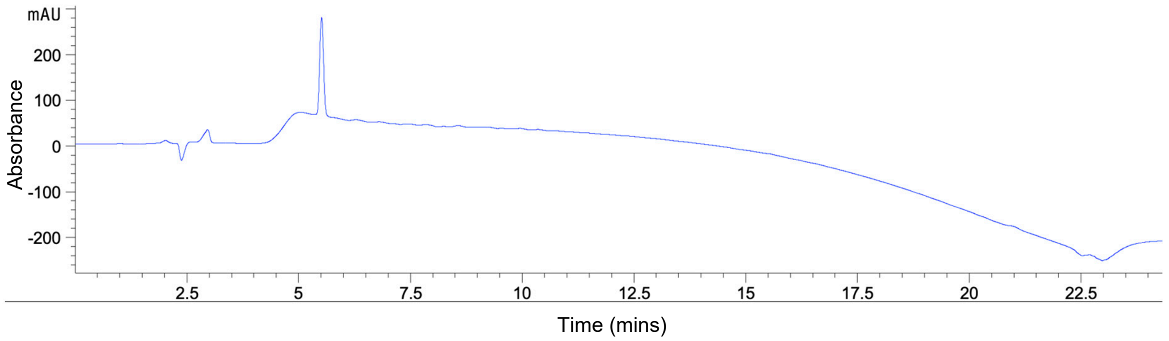

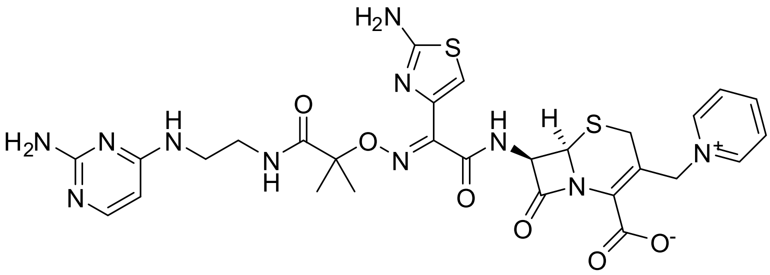

(6R,7R)-7-((E)-2-(((1-((2-((2-aminopyrimidin-4-yl)amino)ethyl)amino)-2-methyl-1-oxopropan-2-yl)oxy)imino)-2-(2-aminothiazol-4-yl)acetamido)-8-oxo-3-(pyridin-1-ium-1-ylmethyl)-5-thia-1-azabicyclo[4.2.0]oct-2-ene-2-carboxylate (14). Prepared in an analogous manner to compound 1. A reaction on a 0.31 mmol (200 mg) scale yielded 14 mg of compound **14** as a white powder (7% yield). ^{1}H NMR (500 MHz, DMSO-d6) 9.62 (1H, d, J = 8.0 Hz), 9.13 (2H, d, J = 5.8 Hz), 8.57 (1H, t, J = 7.6 Hz), 8.12 (2H, t, J = 6.9 Hz), 7.57 (2H, broad s), 7.30-7.12 (6H, m), 6.79 (1H, s), 5.97 (1H, dd, J = 4.75 Hz, 8.13 Hz), 5.61 (1H, d, J = 14.8 Hz), 5.52 (1H, d, J = 14.7 Hz) 5.24 (1H, d, J = 5.08 Hz), 3.56 (1H, d, J = 18.3 Hz), 3.41 (1H, d, 18.2 Hz), 3.26 (2H, m), 1.35 (6H, s), 1.23 (2H, m). ^{13}C NMR (500 MHz, DMSO-d6), 173.7, 169.0, 164.1, 162.9 (2 overlapping carbons), 158.9, 158.8, 158.5, 158.2 146.4, 145.0, 140.6, 129.2, 128.4 (2 overlapping carbons), 120.0, 115.0, 112.7, 110.4, 98.0, 83.4, 60.7, 58.9, 57.6, 37.6, 25.5, 24.5, 24.2. HRMS (ESI^{+}) calculated for $C_{28}H_{31}N_{11}O_6S_2$ ([M+H]$^{+}$) 682.1973: found 682.1962. Retention time 5.36 mins.

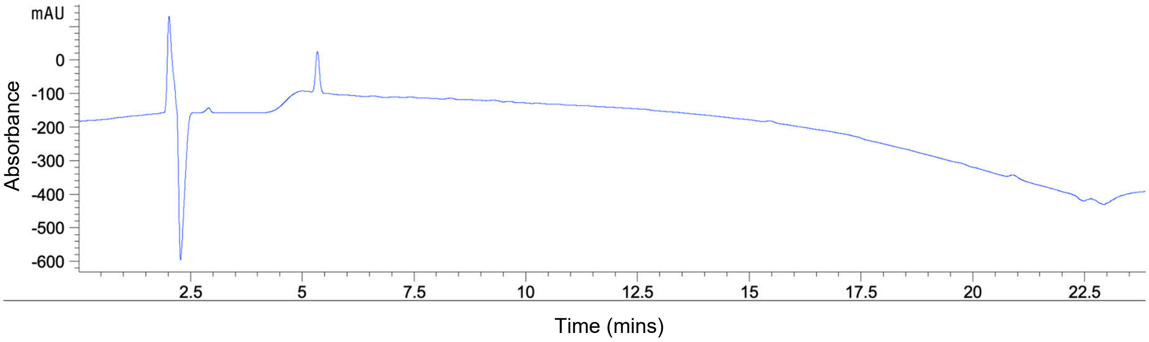

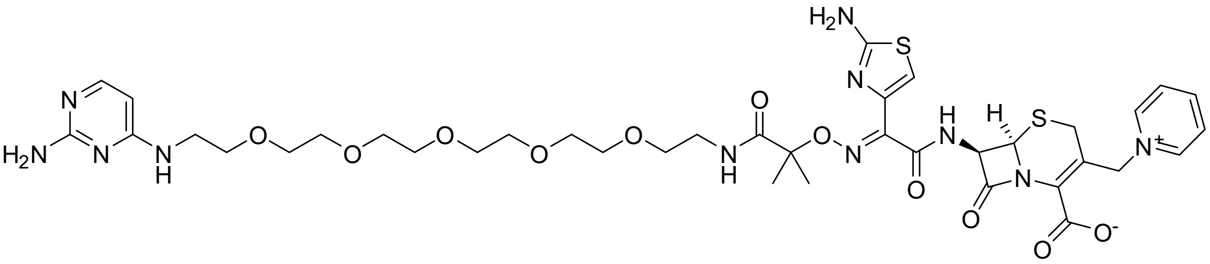

(6R,7R)-7-((E)-24-((2-aminopyrimidin-4-yl)amino)-2-(2-aminothiazol-4-yl)-5,5-dimethyl-6-oxo-4,10,13,16,19,22-hexaoxa-3,7-diazatetracos-2-enamido)-8-oxo-3-(pyridin-1-ium-1-ylmethyl)-5-thia-1-azabicyclo[4.2.0]oct-2-ene-2-carboxylate (15). Prepared in an analogous manner to compound 1. A reaction on a 0.31 mmol (200 mg) scale yielded 11 mg of compound **15** as a white powder (4 % yield). ^{1}H NMR (500 MHz, DMSO-d6) 9.71 (1H, d, J = 8.1 Hz), 9.04 (2H, d, J = 5.7 Hz), 8.66 (1H, t, J = 7.8 Hz), 8.20 (2H, t, J = 7.3 Hz), 7.90 (2H, broad, s), 7.36 (2H, broad s), 7.17 (1H, t, J= 5.9 Hz), 6.80 (1H, s), 6.10 (1H, dd, J = 3.0 Hz, 7.3 Hz), 5.96 (1H, t, J = 5.2 Hz), 5.54 (2H, dd, J = 13.7 Hz, 44.8 Hz), 5.24 (1H, d, J = 4.0 Hz), 3.30-3.60 (28H, m), 1.38 (6H, s). ^{13}C NMR (500 MHz, DMSO-d6), 173.6, 168.8, 164.1, 162.9 (2 overlapping carbons), 146.3, 145.0, 141.0, 129.3, 128.4 (2 overlapping carbons), 120.0, 109.9, 98.0, 83.3, 69.7 (14 overlapping carbons), 68.8, 68.3, 60.7, 58.3, 57.5, 38.7, 25.4, 24.6, 24.1. HRMS (ESI^{+}) calculated for $C_{38}H_{51}N_{11}O_{11}S_2$ ([M+H]$^{+}$) 902.3289; found 902.3354. Retention time 6.68 mins.

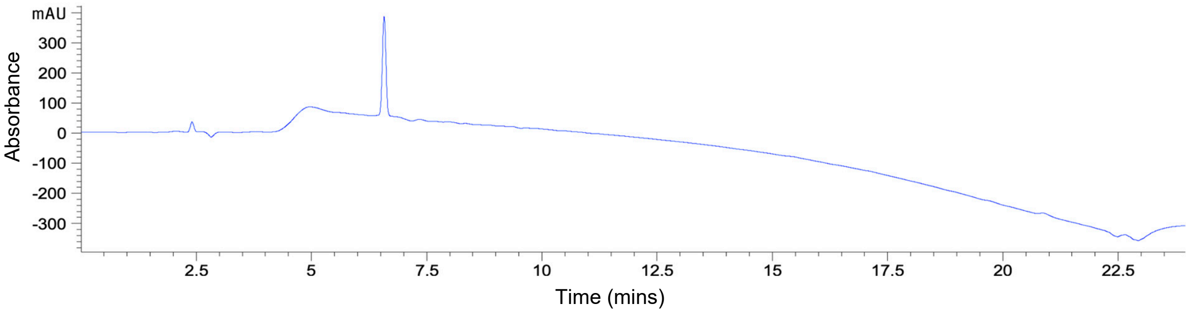

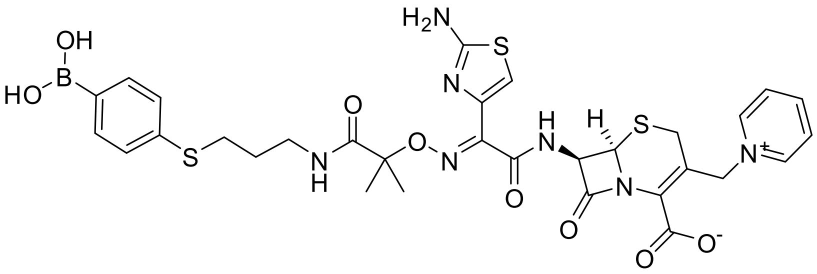

(6R,7R)-7-((E)-2-(2-aminothiazol-4-yl)-2-(((1-((3-((4-boronophenyl)thio)propyl)amino)-2-methyl-1-oxopropan-2-yl)oxy)imino)acetamido)-8-oxo-3-(pyridin-1-ium-1-ylmethyl)-5-thia-1-azabicyclo[4.2.0]oct-2-ene-2-carboxylate (16). Prepared in an analogous manner to compound 1**.** A reaction on a 0.31 mmol (200 mg) scale yielded 24 mg of compound **16** as a light-yellow solid (10.46 % yield). ^{1}H NMR (500 MHz, DMSO-d$_6$) δ 9.75 (1H, d, J = 8.1 Hz), 9.07 – 9.01 (2H, m), 8.64 (1H, tt, J = 7.7, 1.4 Hz), 8.24 – 8.16 (2H, m), 7.73 – 7.67 (2H, m), 7.19 (2H, d, J = 8.1 Hz), 6.81 (1H, s), 5.99 (1H, dd, J = 8.2, 4.9 Hz), 5.62 (1H, d, J = 14.8 Hz), 5.53 (1H, d, J = 14.7 Hz), 5.26 (1H, d, J = 5.0 Hz), 3.54 (1H, s), 3.42 (1H, s), 3.19 (2H, dd, J = 9.7, 6.3 Hz), 2.91 (2H, dd, J = 8.4, 6.4 Hz), 1.73 – 1.65 (2H, m), 1.41 (6H, d, J = 1.5 Hz).^{13}C NMR (126 MHz, DMSO-d$_6$) δ 174.2, 169.4, 164.5, 163.3, 158.9, 158.7, 146.7, 145.2 (2 overlapping C), 139.2 (2 overlapping C), 135.2 (2 overlapping C), 129.7, 128.8 (2 overlapping C), 126.4, 120.4, 117.8, 115.5, 110.7, 84.0, 61.1, 59.3, 57.9, 49.2, 38.2, 29.1, 28.9, 25.1, 24.5. HRMS (ESI$^+$) calculated for $C_{31}H_{34}BN_7O_8S_3$ ([M+H]$^+$) 740.1797; found 740.18072. Retention time 7.61 min.

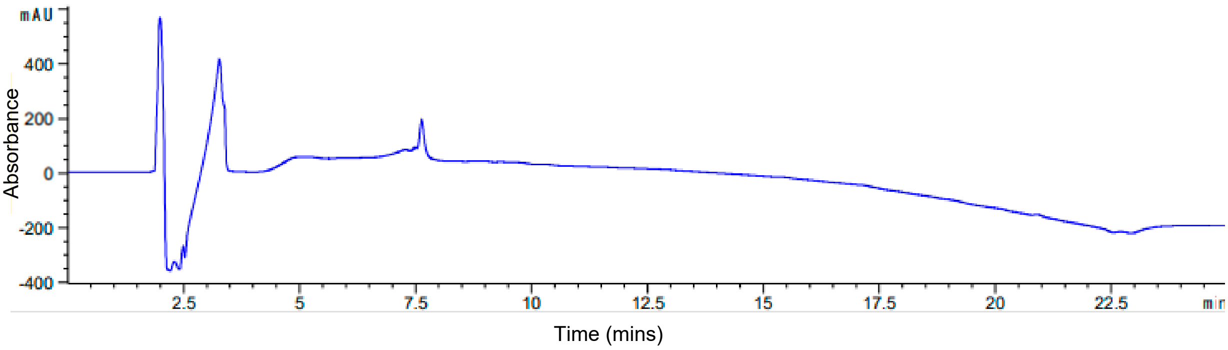

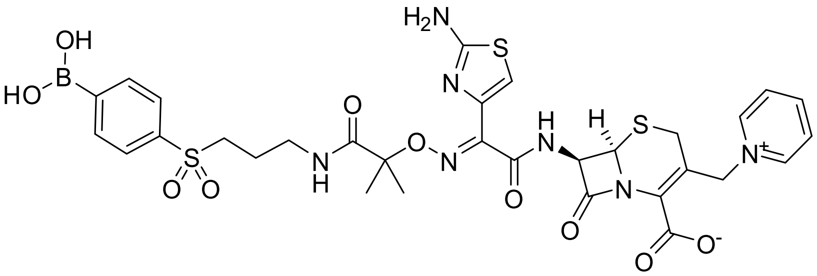

(6R,7R)-7-((E)-2-(2-aminothiazol-4-yl)-2-(((1-((3-((4-boronophenyl)sulfonyl)propyl)amino)-2-methyl-1-oxopropan-2-yl)oxy)imino)acetamido)-8-oxo-3-(pyridin-1-ium-1-ylmethyl)-5-thia-1-azabicyclo[4.2.0]oct-2-ene-2-carboxylate (17). Prepared in an analogous manner to compound 1 A reaction on a 0.31 mmol (200 mg) scale yielded 28 mg of compound **17** as a light yellow solid (11.7 % yield). ^{1}H NMR (500 MHz, DMSO-d$_6$) δ 9.79 – 9.71 (1H, m), 9.02 (2H, dd, J = 10.3, 5.9 Hz), 8.64 (1H, t, J = 7.8 Hz), 8.17 (2H, q, J = 7.1 Hz), 7.43 – 7.38 (2H, m), 7.10 (1H, d, J = 6.6 Hz), 6.95 – 6.88 (2H, m), 6.00 (1H, dd, J = 8.3, 5.0 Hz), 5.65 – 5.62 (1H, m), 5.45 (1H, s), 5.30 – 5.23 (1H, m), 5.19 (1H, s), 3.21 (2H, dq, J = 21.6, 6.9 Hz), 2.74 (2H, dddd, J = 23.7, 19.1, 12.1, 7.2 Hz), 1.57 (2H, dd, J = 15.2, 7.9 Hz), 1.41 (6H, d, J = 15 Hz). ^{13}C NMR (126 MHz, DMSO-D$_6$) δ 173.9, 168.8, 163.3, 160.4, 158.9, 158.7, 146.7, 145.4, 145.2, 133.0, 128.8 (2 overlapping C), 128.6 (2 overlapping C),126.6, 126.6, 126.5 (2 overlapping C), 117.3, 116.5, 115.0, 84.0, 64.4, 61.1, 60.6, 59.3, 53.8, 50.8, 25.3, 24.4, 22.5. HRMS (ESI$^+$) calculated for $C_{31}H_{34}BN_7O_{10}S_3$ ([M+H]$^+$) 772.1695 amu, but observed without boronic acid as $C_{31}H_{33}N_7O_8S_3$ ([M+H]$^+$) calculated 728.1600 found 728.15922 amu. MALDI-TOF calculated for $C_{31}H_{34}BN_7O_{10}S_3$ ([M+Na]$^+$) 794.1515; found 794.291. Retention time 6.45 min.

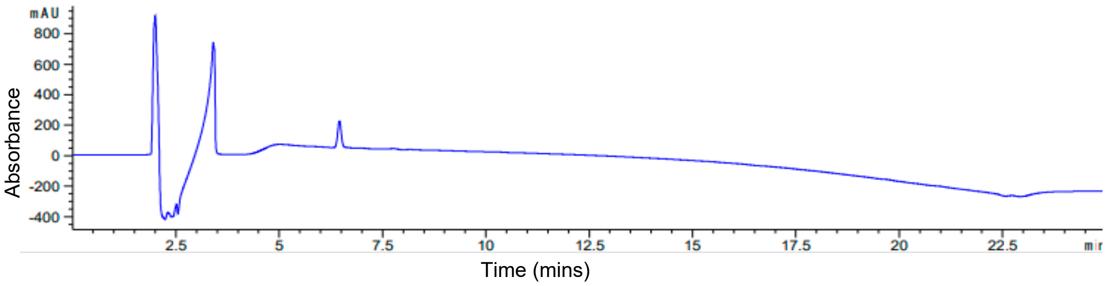

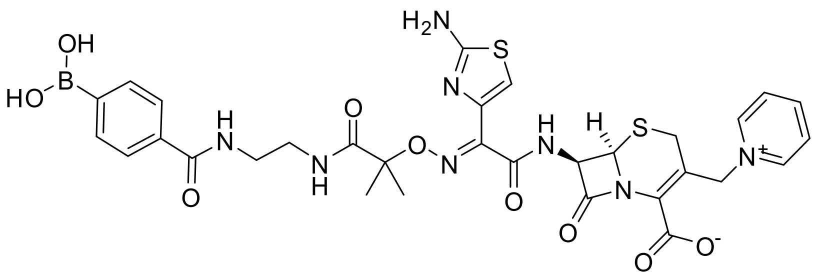

(6R,7R)-7-((E)-10-(2-aminothiazol-4-yl)-1-(4-boronophenyl)-7,7-dimethyl-1,6-dioxo-8-oxa-2,5,9-triazaundec-9-en-11-amido)-8-oxo-3-(pyridin-1-ium-1-ylmethyl)-5-thia-1-azabicyclo[4.2.0]oct-2-ene-2-carboxylate (18). Prepared in an analogous manner to compound 1: A reaction on a 0.31 mmol (200 mg) scale yielded 15 mg of compound **18** as a light yellow solid (6.56 % yield). ^{1}H NMR (500 MHz, DMSO-d_6) δ 9.43 (2H, d, J = 9.0 Hz), 9.12 (1H, d, J = 6.0 Hz), 8.59 (1H, d, J = 9.1 Hz), 8.18 – 8.13 (2H, m), 7.28 (2H, s), 6.82 (1H, s), 5.74 (1H, dd, J = 8.3, 4.9 Hz), 5.64 (2H, dd, J = 22.0, 13.4 Hz), 5.45 – 5.43 (1H, m), 5.32 (1H, d, J = 3.9 Hz), 5.10 (2H, d, J = 5.0 Hz), 4.24 (1H, s), 3.81 (1H, d, J = 5 Hz), 3.53 (2H, s), 1.42 (6H, d, J = 1.8 Hz).^{13}C NMR (126 MHz, DMSO-d_6) δ 175.4, 168.9, 167.6, 163.4, 162.4, 150.1, 149.7, 149.7, 146.4, 146.1, 145.6, 145.4 (2 overlapping C), 143.1, 138.4, 136.6, 128.5 (2 overlapping C), 128.4, 124.4 (2 overlapping C), 122.9, 121.8, 110.4, 110.2, 82.2, 64.7, 62.0, 60.1, 57.9, 53.8, 24.7, 24.3. HRMS (ESI^{+}) calculated for $C_{31}H_{33}BN_8O_9S_2$ ([M+H]$^{+}$) 737.1978; found 737.1965. Retention time 5.95 min.

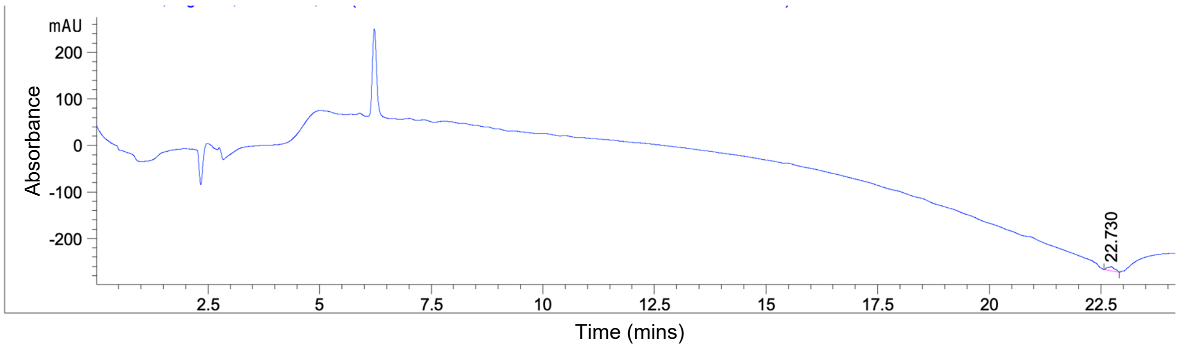

Supplement: Supplementary file 1 [file antibiotics-14-00177-s001.zip › antibiotics-3422488-supplementary.pdf]
